# Hybrid and conjugated antimicrobial peptides: new tactics to counter bacterial resistance

**DOI:** 10.3389/fmicb.2026.1670569

**Published:** 2026-03-10

**Authors:** Yuanyuan Zhang, Cui Bao, Jiao Feng, Xiuwen Hong, Nan Gao, Ganzhu Feng

**Affiliations:** 1 Nanjing Medical University, Nanjing, China; 2 Department of Respiratory and Critical Care Medicine, The Second Affiliated Hospital of Nanjing Medical University, Nanjing, China

**Keywords:** antibiotic, antimicrobial peptides, conjugation, drug resistance, hybridization

## Abstract

The phenomenon of bacterial resistance has emerged as a significant challenge to global public health. Due to the increasing prevalence of antibiotic resistance, there has been interest in developing antimicrobial peptides (AMPs) as alternative antimicrobial therapies. However, AMPs resistance is not uncommon; it is simply subject to complex ecological and physiological limitations. While AMPs demonstrate potent antimicrobial activity in experimental and preclinical studies, their clinical efficacy remains limited. This review mainly summarizes the two methods of peptide hybridization and conjugation to combat drug-resistant bacteria. Hybridization has given AMPs new vitality, which overall enhance their antimicrobial spectrum, reduce toxicity, and enhance the bactericidal effect on drug-resistant strains. We also reviewed the conjugation of AMPs with various active molecules, such as antibiotics, antibodies, fatty acids, photosensitizers, phosphodiester oligomers, and nanoparticles. This review provides ideas for the design of hybrid peptides and coupled peptides in the future, and these AMPs have been shown to have an effect on drug-resistant strains after hybridization or coupling, thereby making the originally ineffective AMPs regain sensitivity. The transformation of natural AMPs has been effective in the laboratory to some extent, and give it clinical exploration value. Their clinical performance still falls short of that of conventional antibiotics due to challenges related to pharmacokinetics, safety, and reduced activity under clinically relevant conditions. To break through the bottleneck of clinical transformation of AMPs, it is necessary to continue to deepen multi-dimensional research on their physicochemical properties and make good use of artificial intelligence technology for intelligent design and high-throughput verification of hybrid peptides or conjugated peptides.

## 1 Introduction

Humans have utilized antimicrobial agents for thousands of years, with ancient Chinese employing herbs to combat bacterial infections. The 20th century witnessed a landmark breakthrough with the discovery of penicillin, marking a pivotal moment in antimicrobial development. Since then, antimicrobial drugs have proliferated significantly (Gan et al., 2021). According to “WHO releases new reports on new tests and treatments in development for bacterial infections (2025),” seventeen new antibacterial agents against priority bacterial pathogens have obtained marketing authorization since July 2017, but only two represent a new chemical class. The pipeline now faces a dual crisis: scarcity and lack of innovation. Meanwhile, the emergence of antimicrobial resistance is accelerating at a pace that outstrips current drug discovery capabilities. Alarmingly, new drugs often encounter resistance even before their commercial release (Gray and Wenzel, 2020).

The resilience of bacteria arises from multiple adaptive mechanisms, namely enzymatic inhibition, biofilm coverage, porin mutations, efflux pumps and molecular modifications of antibiotic targets (Sadeer and Mahomoodally, 2021). Traditional antibiotics such as fluoroquinolones, aminoglycosides, and β-lactams, target specific cellular components and susceptible to mutation, and are primarily effective against metabolically active bacteria but ineffective against dormant persister cells (Gan et al., 2021). Collectively, these strategies have severely undermined the efficacy of conventional antibiotics and underscored the urgent need for alternative therapeutic paradigms. Natural antimicrobial peptides (AMPs) have emerged as promising candidates. Acting often through disruption of bacterial membranes, AMPs compromise the structural integrity of the phospholipid bilayer, leading to its death (Yuan et al., 2025). On the one hand, the high metabolic cost required for bacterial membrane repair imposes a significant metabolic burden, thereby limiting but not precluding the stable evolution of resistance to AMPs (Fadaka et al., 2021). On the other hand, certain AMPs can also act on dormant bacteria (Mohammadi et al., 2025).

However, resistance to antimicrobial peptides has also emerged. AMP-resistant bacteria employ diverse mechanisms, including but not limited to proteolytic degradation, extracellular trapping and inactivation, active efflux, as well as complex modifications in bacterial cell wall and membrane structures (Tajer et al., 2024; Andersson et al., 2016). Another problem currently faced is their systemic toxicity, susceptibility to protease degradation, short half-life, low target specificity, high production costs and rapid renal clearance. Their clinical application is limited to local application (Fadaka et al., 2021; Silva et al., 2022). Therefore, it needs to be transformed.

The hybrid peptides refer to AMPs that contain fusions of two or more naturally occurring peptide sequences to provide bifunctional properties, which is expected to increase potency, enhance selectivity, reduce cytotoxicity, or produce a dual mode of action (Gan et al., 2021; Zhang et al., 2018; Pedron et al., 2023). Conjugation to molecules such as polymers, small molecules, and nanoparticles may increase the therapeutic potential of AMPs, improve stability, reduce toxicity, and reduce susceptibility to proteolytic attack (Silva et al., 2022). There are abundant sources of natural peptides, which can provide rich resources for hybridization or conjugation.

## 2 Antimicrobial peptide source

Natural AMPs in the APD3s (accessed on 12 July 2025)^1^ are classified into six life kingdoms: bacteria (410), archaea (prokaryotes) (5), protists (8), fungi (29), plants (268), and animals (eukaryotes) (2580) (Wang et al., 2016). The sources, classifications and examples of AMPs are listed in Table 1. The human microbiota and microbiome are hosts to important peptides. The human microbiome is largely composed of bacteria, including major phyla such as Firmicutes, Bacteroidetes, Actinobacteria, Proteobacteria, and Fusobacteria. Peptides extracted from these bacteria are the primary agents exhibiting high antimicrobial activity and have been demonstrating this activity for decades. Biosynthetic Gene Cluster (BGC) mining and AI prediction tools (such as antiSMASH and DeepRiPP) can systematically identify potential anti-resistance peptides, providing a crucial molecular resource for future anti-resistance strategies (Shah et al., 2025; Shah and Shim, 2025).

**TABLE 1:** The sources, classifications and examples of AMPs.

Source	Classification	References	Description	Example
Phage	Lysins	Sabur et al., 2025	More effective against Gram-positive bacteria than Gram-negative bacteria	LysMR-5
	VAPGHs	Gutiérrez et al., 2018	Participate in the initial steps of infection by slightly degrading peptidoglycan to allow entry of the phage genetic material into the bacterial cell	HydH5
	Depolymerases	Knecht et al., 2019	Specificly bind to capsular polysaccharides (CPS), exopolysaccharides (EPS) or lipopolysaccharide (LPS) of the host bacteria	K1
	Holins	Bruser and Mehner-Breitfeld, 2022	Generate large membrane lesions that permit the passage of endolysins across the cytoplasmic membrane of prokaryotes	HolGH15
Microorganism	Gram-positive	Simons et al., 2020	Represent the major part of bacteriocins	Nisin
	Gram-negative	Simons et al., 2020	Most of them were isolated from *Escherichia coli* strains	Microcin J25
Fungi	Peptaibols	Hou et al., 2022	Exhibit a wide range of biological activities, including anti-microbial, cytotoxic, and neuroleptic effects	Alamethicin
	Fungal defensins	Silva et al., 2014	Active against predominantly Gram-positive bacteria	Plectasin
Plant	Thionins	Hong et al., 2021	Found only in plants.	CaThi
	Defensins	Lima et al., 2022	Inhibit microbial growth, inhibit α-amylase and trypsin activity	Dm-AMP1
	Hevein-like peptides	Lima et al., 2022	Have a conserved chitin-binding domain and have antifungal activity	Hevein
	Knottins	Li et al., 2021	Have antimicrobial activity against bacteria, fungi, viruses and insects	Ep-AMP1
	Stable-like peptides	Lima et al., 2022	Binds to DNA, inhibit RNA and protein synthesis	–
	Lipid transfer proteins	Lima et al., 2022	Inhibit the growth of fungi and bacteria, and participate in plant defense systems	CmLTP
	Snakins	Lima et al., 2022	Inhibits the growth of certain bacteria and fungi	Snake -1
	Cyclotides	Yuan et al., 2025	Cytotoxic, inhibits cancer, membrane-penetrates, induces endocytosis.	MCoTI-II
Animal	Invertebrates	Brady et al., 2019	Promising antibacterial therapeutic candidates, including low toxicity against mammalian cells and anti-inflammatory activity	Cecropins
	Fish and amphibians	Wang, 2020; Mangoni and Casciaro, 2020	Actually the most numerous and the most studied	Ranalexin
	Reptiles and birds	van Hoek, 2014	Express major classes of antimicrobial peptides	OH-CATH
	Mammals	Bin Hafeez et al., 2021	Can act directly on pathogens and can also regulate immune responses, apoptosis and wound healing	LL-37

## 3 Factors regulating antimicrobial peptide activity

Antimicrobial peptides exhibit several defining characteristics. First, most are short cationic peptides with a net positive charge typically ranging from +3 to +11. Second, they contain hydrophobic residues that strongly interact with the lipid membrane’s hydrophobic core. Third, in the hydrophobic environment of lipid bilayers, they adopt distinct secondary structures such as α-helices or β-sheets (Islam et al., 2023).

**FIGURE 1:**
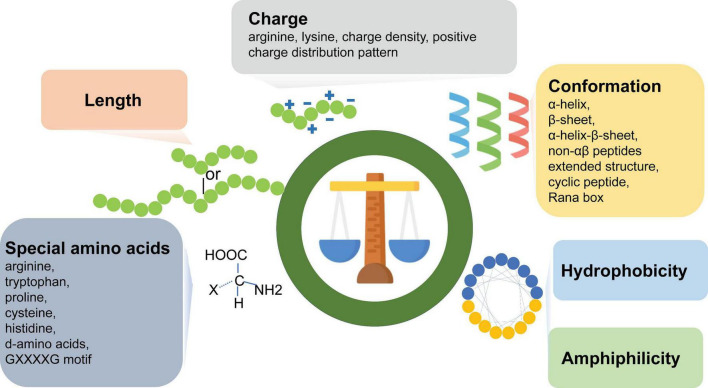
Six key factors regulating antimicrobial peptide activity: peptide length, charge, conformation, hydrophobicity, amphipathicity, and presence of special amino acids. GXXXXG, G, glycine; X, other amino acids.

A significant proportion of natural peptides have failed to demonstrate sufficient antimicrobial activity and pharmacological properties in clinical studies. Their poor performance may stem from the disparity between the complex clinical environment and their natural conditions (Chen and Lu, 2020). To develop AMPs with clinical utility, continuous modification and optimization of natural AMPs remains imperative. The structural and physicochemical parameters that affect the antimicrobial activity and toxicity of AMPs include peptide length, net charge, conformation, hydrophobicity, amphipathicity, special amino acids, etc., (Goki et al., 2024) (see Figure 1). All properties of AMPs, such as charge, hydrophobicity, amphipathicity and α-helicity, are interrelated, and modifying one parameter affects the others, thereby altering the overall properties and antimicrobial activity of the individual peptides (Goki et al., 2024).

### 3.1 Length

Gagnon et al. (2017) synthesized a series of AMPs with the repeating sequence KIAGKIA as the core, ranging from 14 to 28 amino acids in length, with a constant charge (+7). They found that the antibacterial activity, hemolysis and membrane leakage of the peptides all showed obvious length dependence. As demonstrated by leakage experiments, compatibility between peptide length and membrane thickness is a prerequisite for the manifestation of peptide activity. Indeed, at peptide lengths of 19 amino acids or less, hemolysis was almost non-existent even at high concentrations of 512 μg/mL (Gagnon et al., 2017).

### 3.2 Charge

There is a general consensus in the scientific literature that increasing net charge is positively correlated with antibacterial activity. However, once a peptide’s optimal charge threshold has been reached, further charge enrichment increases toxicity rather than antibacterial activity (Tan et al., 2021). There is no consensus on a universal optimal charge threshold, as this varies across AMPs. Instead, an AMP’s optimal charge threshold is often determined empirically. For example, in the 26-residue amphipathic α-helical antimicrobial peptide L-V13K, the optimal number of positively charged residues on the polar surface is seven (net charge +8). Reducing the net charge decreases the therapeutic index by 5–20 times, while increasing the net charge from +8 to +9 significantly increases hemolytic activity by more than 32-fold. This indicates that there is a threshold between increasing net charge and antibacterial activity (Tan et al., 2021; Jiang et al., 2008). Replacing Lys in the parent peptide jelleine-1 and its analogues with Arg increases antibacterial activity by 2–4 times. This is because the physicochemical properties of the side chain functional groups of lysine (imino) and arginine (guanidino) are different. The membrane deformation caused by charged Arg is very similar to that of Lys, but it can attract more lipid head groups (Zhou et al., 2021). In addition to net charge, charge density and charge placement within the AMP can also influence antimicrobial and toxicity properties. Charge density refers to the net charge relative to the number of total residues within the AMP and charge placement is the location of charge within the AMP sequence. Regarding charge density, Islam et al. (2023) found no consistent correlation with antimicrobial activity in their investigation of amphibian AMPs. Whether this applies to AMPs from other sources is unknown. Stone et al. (2019) concentrated the positive charge (Lys) at the N-terminus of AMP 6K-F17 and found that this made it easier to target negatively charged bacterial membranes and improved the protease resistance of the peptides.

### 3.3 Conformation

The secondary structures of AMPs can be classified as α-helix, β-sheet, α-helix-β-sheet, non-αβ peptides or extended structure. For some AMPs, the defined secondary structure does not necessarily play an important role in antimicrobial activity, and only the subtle interaction between positive charge and hydrophobicity is sufficient to produce the desired biological activity. Enhanced helicity can increase the antimicrobial activity of some AMPs (Goki et al., 2024). Cyclic peptides tend to have higher antibacterial activity, stability and longer half-life (Pei et al., 2025). For example, the 10-amino acid residue cyclic peptide from *Jatropha multifida*, Lobaditin (Lo), possesses bactericidal activity against *Staphylococcus aureus*. However, its linear analogue (L1), was inactive against *S. aureus*. The cyclic structure of Lo may provide a rigid conformation that is key for the self-assembly of peptide nanotubes that induce pore formation in large unilamellar vesicles, according to permeability assays and dynamic light scattering measurements (Nobre et al., 2016). Other conformational domains such as the “Rana box” are thought to play important roles in maintaining the activity and stability of AMPs. “Rana box” refers to a special structural domain commonly found in amphibian AMPs (such as Brevinin-like peptides). It is a disulfide bond ring structure formed by two cysteine (C) residues in the C-terminal sequence. The function of this motif varies across different peptide families: removal of the Rana box significantly reduces the bioactivity of peptides in Ranatuerin-2 and Brevinin-1, while the presence of the motif has little effect on peptides in esculentin-1 (Lin et al., 2021).

### 3.4 Hydrophobicity

Comparative analysis of most AMPs reveals that hydrophobic amino acid residues account for approximately 40%–60% of the total amino acid residues (Tan et al., 2021). Kumar et al. (2024) conducted predictions and statistical analyses of physicochemical and structural parameters for a dataset of 3,697 experimentally validated peptides. Their findings indicate that AMP exhibits an average hydrophobicity parameter of 1.2, demonstrating balanced hydrophobicity (Kumar et al., 2024). Wang (2020) performed bioinformatics analysis on 1,000 amphibian antimicrobial peptides and found that the ratio between hydrophobic and charged amino acids is a determinant of the activity spectrum of a peptide.

### 3.5 Amphipathicity

Amphipathicity is quantified by the hydrophobic moment, which is the vector sum of the individual hydrophobic amino acids in the normalized helical structure, with higher values indicating greater amphipathicity. The amphiphilicity of antimicrobial peptides is related to their ability to permeate membranes. Cheng et al. formed an amphiphilic structure by alternating hydrophobic amino acids (Leu, Ile, etc.) and hydrophilic amino acids (Arg, Lys, etc.). The cationic residues electrostatically attract the negatively charged bacterial membrane, while the hydrophobic amino acids insert into the membrane to form pores, ultimately leading to bacterial death (Zeng and Cheng, 2022). He et al. (2020) studied the optimal amino acid arrangement pattern of amphiphilic β-fold antimicrobial peptides and found that a stable amphiphilic β-fold structure is closely related to strong antibacterial activity. Typically, perfect amphiphilic distribution leads to great broad-spectrum antimicrobial activity, simultaneously accompanying increased toxicity against healthy cells (He et al., 2020).

### 3.6 Special amino acids

Mammalian AMPs tend to be enriched in histidine, proline, and arginine, whereas amphibian AMPs often contain higher levels of alanine, glycine, and leucine. Natural AMPs simultaneously enriched in leucine and lysine are rarely reported, whereas peptides rich in proline, tryptophan, and cysteine frequently co-occur with arginine (Decker et al., 2022). Tryptophan is very important for membrane-peptide interactions. The tryptophan side chain can interact with lipids in a variety of ways, including hydrogen bonding, hydrophobicity, π-π, cation-π, and anion-π (Straus, 2024; Le et al., 2015). Cysteine can stabilize β-sheet structures via disulfide bonds, while histidine-rich peptides have been explored as pH-responsive designs (Tan et al., 2021). Glycine can play a key role in transmembrane helical interactions, particularly in double glycine motifs such as GXXG, GXXXG, and GXXXXG (Tripathi et al., 2017). Tripathi et al. (2017) found a critical role of the GXXXXG motif in the modulation cell selectivity of Chrysophsin-1, demonstrating that the replacement of glycine with proline can reduce cytotoxicity while retaining antibacterial and antiendotoxin activities. Indeed, the introduction of proline at the central position of some α-helical AMPs has been shown to improve the selectivity toward bacterial cells compared to Gly or Ala (Yang S. et al., 2022). Proline is commonly known as a helix-breaking amino acid. Some proline-rich antimicrobial peptides have been shown to target ribosomes and interfere with protein synthesis (Graf et al., 2017). Incorporating D-amino acids into the AMP sequence changes the chirality of the amino acids. Because of the mismatch between chirality and the enzyme’s active site, D-enantiomers are more tolerant to endogenous enzymes, especially proteases. Moreover, the replacement of D-amino acids tends to change the structure and amphipathicity without affecting the hydrophobicity and net charge (Jariyarattanarach et al., 2022; Kumar et al., 2018). However, some studies have found that the insertion of glycine, proline, and D-amino acids can destroy the secondary structure of peptides (Dini et al., 2022).

## 4 Hybridization strategies to combat drug resistance

Traditional chemical transformation and modification are based on the six factors mentioned above that affect the activity of AMPs, such as point mutation (Ferreira et al., 2021), post-translational modification or cyclization (Mwangi et al., 2023). This article focuses on the hybrid peptide strategy. Hybridization of peptides is an effective strategy for generating new AMPs that, if designed properly, can minimize cytotoxicity and significantly improve antimicrobial activity (Cheng et al., 2021). This approach typically involves combining two natural AMPs, or combining natural AMPs with other peptides. Hybrid peptides combining different domains would ideally exhibit two modes of action (Guo et al., 2024). Table 2 is a non-exhaustive list of successful examples of hybrid peptides reported in the literature. For a more complete table, please see Supplementary Table 1. Research has focused on hybridization of membrane-permeable AMPs, such as cecropin, melittin, magainin, cathelicidin, aurin, LL-37, and lactoferrin (Trevellin et al., 2024). Most studies designed hybrid peptides by observing their hydrophobicity, amphipathicity, and positive charge, and the best hybrid peptides did not correspond to the optimal parameter values (Le et al., 2015; Yang H. et al., 2022). Peptide hybridization is an underexplored molecular engineering tool that has opened up new avenues for the design of bioactive peptides (Pedron et al., 2023).

**TABLE 2:** Examples of different types of hybrid peptide.

Hybridizer	Source	Modification	Linker	Antibacterial spectrum	Effect of hybridization	References
**Hybridization between two natural antimicrobial peptides**						
Melittin, thanatin	*Apis mellifera*, *Podisus maculiventris*	Truncate melittin (Ala→Lys substitution)	AGP	*E. coli* JM109, *S. aureus*, *Bacillus subtilis*, *Salmonella Typhimurium*	Produce an anti-*S. aureus* effect and reduce hemolysis	Jiang et al., 2019
VmCT1, anoplin/protonectin/ decoralin/temporin A	*Vaejovis mexicanus*, *Anoplius samariensis/Agelaia pallipes/Oreumenes decorates/Rana temporaria*	Truncate the amphipathic segment of the parent peptide	G	*A. baumannii* ATCC 19606, *E. coli* (ATCC 11775, AIC221, AIC222 stains), *Klebsiella pneumoniae* ATCC 13883, *S. aureus* ATCC 12600, and etc.	Redirect biological activity, present increased antimicrobial activity (3.1–128 μmol/L) with almost no adverse toxicity to erythrocytes	Pedron et al., 2023
Papiliocin, magainin II	*Papilio xuthus*, *Xenopus laevis*	Truncate both parent peptide	P/a lysine peptoid analogue	*B. subtilis* KCTC 3068, *E. faecalis* KCTC 2011, *S. aureus* KCTC 1621, *S. typhi* KCTC 1926, *P. aeruginosa* KCTC 1637, *E. coli* KCTC 1682	Have antibacterial and anti-inflammatory activities, PapMA-k display enhanced bacterial selectivity	Shin et al., 2015
Magainin II, cecropin B	*Xenopus laevis,* *Cecropia moth*	Unmodified	Directly linked	*E. coli* ATCC 25922	Display antibacterial and immunomodulatory activities in mice	Zhang et al., 2018
CopA3, Hp1090	*Copris tripartitus*, *Heterometrus petersii*	Unmodified	GG/GGGGGG	*E. coli* (D31 and JM83 strains)	Have more potent bactericidal activity, adjust the nature of the linker peptide to modulate the cytotoxic activity	Tonk et al., 2021
BMAP-28, LL-37	Bovine neutrophils, human	Extract the helical portion of the parent peptide, BMAP-28: Arg3→Lys3, Lys14→Arg14 substitution, LL-37: Phe→Ala, Lys→Val substitution	Directly linked	MRSA, MDR *E. coli*, *S. aureus* (29213 and BAA-41 stains)	Have higher antimicrobial activity	Masadeh et al., 2022
BF2, DesHDAP1	Histone-derived AMPs	Unmodified	A/P/G/hydroxyalanine	*E. coli*, *B. subtilis*	Have higher antimicrobial activity with no cytotoxicity against eukaryotic cells	Trevellin et al., 2024
EnterocinK, EJ97	Bacteriocins	Truncate both parent peptide	YEI	*Staphylococcus haemolyticus*	Differ from other bacteriocins in the inhibition spectrum	Kranjec et al., 2021
Cesin, rombocin	Nisin	Truncate both parent peptide	Directly linked	*Lactococcus lactis* MG1363, *Listeria monocytogenes* LK132, *Bacillus cereus* CH-85, *S. aureus* LMG10147, MASA, *E. faecium (*LMG11423, LMG16003 strains)	Improves stability against trypsin degradation to over 50%, show no undesired toxic effects, remain stable in human plasma	Guo et al., 2024
**Thymosin**						
LL-37(13–36)/YW12D/innate defense regulator 1/cathelicidin 2, TP5/Tα1(17–24)	Human/synthesis/ chicken synthesis/thymic stromal cells	Truncate both parent peptide	Directly linked	Bind to LPS	Enhanced anti-inflammatory activity and reduced cytotoxicity	Zhang et al., 2019
Cathelicidin 2, TP5	Chicken synthesis	Truncate both parent peptide, add Gln to the C-terminus	Directly linked	*S. aureus* ATCC 43300	Have effective antibacterial, antibiofilm, and anti-adhesion activities	Guo et al., 2021
**New amyloidogenic antimicrobial peptides**						
CPP, Amyloidogenic sequence of ribosomal S1 protein of *P. aeruginosa*	Synthesis	Sarcosine12 instead of Gly12	GGGG,GG-Sar-G	*P. aeruginosa* (PA103 and ATCC 28753 stains)	The MIC was comparable to the antibiotic gentamicin sulfate.	Grishin et al., 2021
CPP, Amyloidogenic sequence of ribosomal S1 protein from *S. aureus*	Synthesis	Asi19 instead of Asn19, sarcosine instead of Gly	GG-Sar-G	MRSA (ATCC 43300 strain), *S. aureus* 209P, *E. coli* K12 strain, *B. cereus* IP-5812 strain	Active against Gram-positive and Gram-negative bacteria	Kravchenko et al., 2022
CPP, Amyloidogenic sequence of ribosomal S1 protein from *S. aureus*	Synthesis	Replacement of canonical amino acid residues with other canonical or non-canonical amino acid residues (X)	GG-Sar-G	*S. aureus* (209P and 129B strains), MRSA (SA 180 and ATCC 43300 strains), *B. cereus* (strain IP 5832), *P. aeruginosa* (ATCC 28753 and 2943 strains) *E. coli* (MG1655 and K12 strains)	Exhibit antimicrobial activity comparable to gentamicin and meropenem	Kravchenko et al., 2024
**Targeting peptides**						
An LPS binding sequence, PMAP-23	Porcine neutrophils	Arg instead of Asp	PQKP	*E. coli* ATCC 25922, *S. aureus* (ATCC 25923 and 29213 strains), *S. typhi* C7731, *S. epidermidis* ATCC 12228	Enhance LPS affinity	Lyu et al., 2023
A targeting peptide binds to *P. aeruginosa*, GNU7	Synthesis	Unmodified	GGG	MDR *P. aeruginosa*	Exhibit a high degree of specificity for *P. aeruginosa*	Kim et al., 2020

A, alanine; G, glycine; P, proline; S, serine; Q, glutamine; K, lysine; Y, tyrosine; E, glutamic acid; I, isoleucine.

### 4.1 Hybridization between two natural antimicrobial peptides

Hybridization of different AMP can create novel peptides with specific properties (Guo et al., 2024), generate more positive charges and increase helicity (Masadeh et al., 2022), can sometimes cause two peptides that do not inherently possess antimicrobial activity to exhibit antimicrobial effects after hybridization (Serafin et al., 2023). Effective hybridization does not necessarily require the two antimicrobial peptides to have the same mechanism of action, for example, the hybridization of membrane-translocating AMPs may ultimately lead to membrane permeability, possibly due to changes in specific physicochemical properties (Trevellin et al., 2024). Le et al. (2015) focused on hydrophobicity and net charge in the design of five hybrid peptides based on Indolicidin and Ranalexin and the hybrid peptides RN7-IN10, RN7-IN9, RN7-IN8, and RN7-IN6 exhibited significant antibacterial activity with MIC values of 7.81–15.62 μg/ml against *Streptococcus pneumoniae*, which were lower than parent peptides indolicidin (MIC = 15.62–31.25 μg/ml) and ranalexin (62.5 μg/ml). Other hybridization approaches may focus on limiting toxicity or increasing antimicrobial activity. For example, Jiang et al. (2019) designed a hybrid peptide comprising melittin and thanatin, in which thanatin exhibited no inhibitory activity against *S. aureus*. The clinical application of Melittin is limited due to its strong toxicity (mainly hemolytic activity), genotoxicity and effects on gene expression. The hybrid peptide demonstrated potent antibacterial activity against *S. aureus* (MIC of 1.2–2.5 μmol/L) similar to melittin (MIC of 0.9–1.5 μmol/L), and the hemolytic concentration was greater than 45 μmol/L. Structural analysis demonstrated that the α-helical fragment of Melittin and the β-layer fragment of Thanatin in the hybrid peptide maintained their independence in spatial structure and did not interfere with each other, thereby preserving Melittin’s capacity to inhibit *S. aureus*. Therefore, the hybridization of two natural AMPs can compensate for the deficiencies of the parent peptides, thereby constructing an AMP with enhanced efficacy.

Despite the numerous documented instances of successful hybridization designs, the experimental conditions remain comparatively restricted, impeding comprehensive prediction of these peptides’ performance within complex biological systems. Furthermore, most hybrid peptides are currently designed artificially, which may lead to ineffectiveness after hybridization, resulting in a high failure rate.

### 4.2 Hybridization of natural antimicrobial peptides with other peptides

#### 4.2.1 Thymosin

Thymopentin (TP5) is a synthetic peptide composed of 5 amino acid residues, and thymosin α 1 (Tα1) is a 28 amino acid peptide produced by thymic stromal cells. Furthermore, TP5 and Tα1 have been demonstrated to exhibit moderate immunomodulatory activity and low cytotoxicity. Therefore, they are frequently employed in clinical settings for the treatment of various types of inflammatory diseases. Some AMPs also possess potent immunomodulatory activity relative to that of TP5 and Tα1, yet their cytotoxicity impedes their clinical use. Zhang et al. (2019) designed eight hybrid peptides by combining the active centers of various AMPs (such as LL-37, YW12D, IDR-1, and CATH2) with fragments of TP5 or Tα1. The engineered hybrid peptide LL-37-Tα1 has been shown to enhance the expression of intestinal tight junction proteins, including Occludin and ZO-1, while concomitantly reducing the production of inflammatory mediators such as TNF-α, IFN-γ, IL-6, and IL-1β (Zhang et al., 2019). Cheng et al. (2021) combined three peptide fragments, CM4 (1–8 fragment), LL37 (17–30 fragment) and TP5 (1–5 fragment), to form a hybrid peptide CLP, and efficiently expressed CLP in *E. coli* using the SUMO fusion expression system. The peptide CLP showed potent antibacterial activity against both Gram-positive and Gram-negative bacteria, with an MIC range of 2–8 μg/mL, which was significantly better than the three parent peptides (MIC range of 8–512 μg/mL). Cytotoxicity of CLP also appears to be improved as hemolysis of sheep erythrocyte cells was less than 6.6% at 100 μg/mL, compared with about 30% for human erythrocytes at 90 μg/mL for LL37 (Cheng et al., 2021).

#### 4.2.2 Targeting peptides

Generally speaking, Specific targeted antimicrobial peptides (STAMPs) consist of three parts: a targeting domain, an antimicrobial domain, and a linker (Yang H. et al., 2022). Targeting peptides bind to pathogens by targeting specific determinants on the pathogen surface (e.g., membrane hydrophobicity, charge, pheromone receptors, cell wall components, or characteristic virulence properties), thereby conferring selectivity to the antimicrobial domain. Although a species-specific targeting peptides are difficult to design, the utilization of targeting AMPs as a substitute for traditional antibiotics is a concept that holds considerable promise (Kim et al., 2019). Which specific components of the bacterial membrane can STAMPs interact with, or whether they exert their antibacterial effects through non-membrane-disrupting mechanisms, requires further investigation (Tan et al., 2021).

Xu et al. (2020) hybridized the *Enterococcus faecalis*-specific pheromone cCF10 as a target domain with the active site of the broad-spectrum antimicrobial peptide C6, and optimized the specificity by reducing the positive charge. The hybrid peptide cCF10-C4 showed highly specific antibacterial activity against *E. faecalis* with a MIC of 8 μM, which was 8 times more active than the parent peptide C4, while it had no significant inhibitory effect on *Staphylococcus aureus*, *Staphylococcus epidermidis*, or all tested Gram-negative bacteria. Notably, cCF10-C4 induced minimal or no hemolysis against human red blood cells at antimicrobial levels.

Antimicrobial peptides can also hybridize with Lipopolysaccharide (LPS) binding sequences. LPS only exists in the outer membrane of Gram-negative bacteria. The binding of AMPs to LPS can reduce the aggregation of LPS and neutralize the toxicity of LPS, but it can also hinder the insertion and transport of AMPs, which may have a negative impact on bactericidal activity. Lyu et al. (2023) used a hinge structure (PQKP) as a linker in the middle of the natural porcine antimicrobial peptide PMAP-23, and the C-/N-terminal sequence was replaced by the LPS binding motif GWKRKRFG. The presence of LPS not only induced the secondary structure of the hybrid peptide to transform from a random structure to an α-helical structure, but also showed excellent binding ability to LPS, which can neutralize LPS and the therapeutic index increased by 8 times (Lyu et al., 2023).

#### 4.2.3 Cell penetrating peptides

Cell penetrating peptides (CPPs) are peptides that can carry substances into cells, similar to AMPs, typically interact strongly with membranes and are cationic and amphipathic. Although CPPs are generally less hydrophobic than AMPs, there is no strict distinction between the two classes of peptides. Many AMPs have been shown to be able to cross bacterial membranes in a manner similar to CPPs and exert antimicrobial activity by binding to intracellular targets, and some CPPs have antimicrobial effects (Deshayes et al., 2017). Classic examples of CPPs include the Tat peptide derived from the HIV-1 Tat protein, penetratin derived from the third helix of the Antennapedia homeodomain protein, and oligomers of arginine (Zeiders and Chmielewski, 2021). Li et al. (2018) conjugated two CPPs (bLFcin6 and Tat11) to the marine AMP N2 and tested the antibacterial effects of these conjugates against intracellular *Salmonella typhimurium*. After 0.5 h incubation, the cell internalization ratio of B6N2 and T11N2 exceeded 28.3% and 93.5%, respectively, which was higher than that of N2 (17.3%). At the same time, the bacterial kill rate increased by 20%. Kravchenko et al. (2023) linked the TAT and Antp fragments of CPPs to the N-terminus and C-terminus of the amyloid peptide, respectively. They also synthesized a two-hybrid peptide containing the TAT fragment at the N-terminus and the Antp fragment at the C-terminus of the amyloid peptide. They found that the TAT-AMP, at a concentration of 12 μM, effectively inhibited *E. coli* growth similar to 200 μM gentamicin.

#### 4.2.4 New amyloidogenic antimicrobial peptides

Some amyloidogenic peptides specifically bind to bacterial ribosomal S1 proteins, induce their co-aggregation and inactivation, thereby blocking key physiological processes of bacteria (Grishin et al., 2021). Known amyloidogenic peptides include Aβ (1–42), serum amyloid A, micromycin E492, and PG-1 (Galzitskaya et al., 2022). Hybrid amyloidogenic antimicrobial peptides (AAMPs) refer to the combination of antimicrobial peptides and amyloidogenic peptides. Hybridization methods have broadened the repertoire of AAMPs.

Galzitskaya et al. (2022) designed hybrid peptides containing three functional parts, namely, cell penetrating peptide, linker, and amyloidogenic region. The CPP utilized TAT peptide and the linker used GGSarG or GGGG sequence. The amyloid regions of the ribosomal S1 proteins of four organisms, *Thermomyces thermophilus* (A), *E. coli* (B), *P. aeruginosa* (C), and *S. aureus* (D), were predicted using four programs: FoldAmyloid, Waltz, acican, and PASTA 2.0. The AAMP constructed based on these regions has a MIC comparable to gentamicin against a variety of pathogenic microorganisms. It is noteworthy that when additional CPPs are introduced into the peptide chain, the peptide chain is lengthened, which appears to reduce its tendency to form amyloid structures (Kravchenko et al., 2023).

### 4.3 Connector

Direct hybridization of two polypeptides may lead to misfolding and damage biological activity. Therefore, it is very important to choose a suitable linker. If the two polypeptides need structural flexibility, flexible linkers such as (GGGGS)n and G(n) can be selected; if the two polypeptides need to ensure the separation of domains, rigid linkers such as α-helix forming linkers (EAAAK)n and proline-rich linkers (XP)n should be selected (Yang H. et al., 2022).

Shin et al. (2015) designed PapMA and PapMA-k, an 18-residue hybrid peptide containing N-terminal residues 1 ∼ 8 of papiliocin and N-terminal residues 4 ∼ 12 of magainin II, connected by proline hinge or lysine-like peptoid, respectively. The structural flexibility of the PapMA-k peptide containing the peptoid can facilitate penetration into the bacterial cell membrane and accumulation in the cytoplasm. In contrast, PapMA shows a rigid curved structure conferred by proline and cannot penetrate the bacterial cell membrane. Yang H. et al. (2022) designed a series of hybrid peptides composed of antimicrobial peptide Ce(1–8) and LPS targeting peptide Lf(28–34) connected by different linkers. The peptide CL1, obtained by direct linking Ce(1-8) and Lf(28-34), showed no activity against most tested Gram-negative bacteria. The flexible linker G4S provided some interdomain movement, leading to interference between the two functional domains and resulting in almost no inhibition against all Gram-negative bacteria. For the peptide CL5 with the rigid linker leucine-proline, the MIC concentration against the tested Gram-negative bacteria was 4–32 μM, representing a 2–16 fold increase in activity compared to Ce(1-8). Different hybrid peptides have different requirements for the linker type, which may be related to the amino acid composition, distribution and properties of the functional domains. By adjusting the repeating units of the linker motif, the length of the linker can be controlled to meet the optimal distance between the functional domains.

## 5 Coupling strategies to combat drug resistance

**FIGURE 2:**
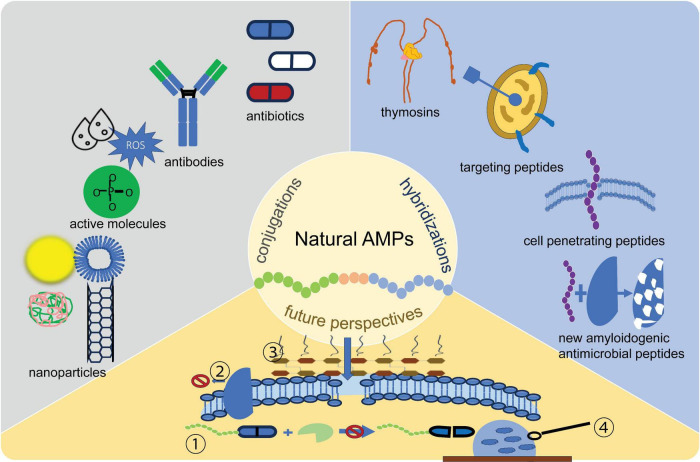
Different hybridizations and conjugations. In the future, it may be possible to develop conjugates coupled with ➀ drug-resistance enzyme inhibitors ➁ efflux pump inhibitors ➂ outer membrane permeabilizers ➃ biofilm inhibitors. AMP, antimicrobial peptides; ROS, reactive oxygen species.

Unlike hybridization, which combines peptide domains within a single sequence, conjugation focuses on attaching antimicrobial peptides to non-peptidic bioactive molecules such as antibiotics, antibodies, small active molecules, or nanoparticles. Different hybridization and coupling types are shown in Figure 2. Examples of antimicrobial peptide conjugates and their mechanisms of action are shown in Table 3.

**TABLE 3:** Examples of antimicrobial peptide conjugates and their combined mechanisms of action.

Conjugate	Modification	Linker	Antibacterial spectrum	Action mechanism	References
**Antibiotics**					
Tobramycin, Bac7 (1–35)	Truncate the parent peptide	Disulfide bond	*E. coli*, *A. baumannii*, *S. enteritidis*, *P. aeruginosa*	Single antibacterial components are released in the bacterial cytoplasm and all target the ribosomes.	Gambato et al., 2023
Tobramycin, CPP	Unmodified	Amide bond or linked by “click” chemistry	*E. coli* MG1655, *S. aureus* S113	Improve the membrane permeability of antibiotics; Reduce the excretion of antibiotics	Deshayes et al., 2017
Penicillin G/Ampicillin, e-poly-1-lysine	Unmodified	Covalent coupling	*E. coli* ATCC 35218, *S. aureus* ATCC 29213	Introduce membrane disruption mechanisms to restore the sensitivity of conventional antibiotics to drug-resistant pathogens	Chen et al., 2024
Ampicillin, 9P2-2	C-terminal modification	Disulfide bond or thioether bond	*E. coli* BW 25113, *A. baumannii* ATCC 19606, *S. epidermidis* ATCC 12228	Penetrating the outer membrane barrier of Gram-negative bacteria (such as *A. baumannii*, *E. coli*)	Yamauchi et al., 2022
Meropenem, Tilapia Piscidin-4	Substitute with D-type amino acid	Lysine residue	*E. coli*, *A. baumannii*, *S. aureus*	Bind to LPS and inhibit the NDM-1 β-lactamase	Selvaraj et al., 2024
Levofloxacin, NST-2 peptide derived from temporin-SHa	Lys4 instead of Gly4, D-alanine substitution at position 10	β-Alanine	*S. aureus* NCTC 13277, *B. subtilis* ATCC 23857, *S. typhi* ATCC 14028, *E. coli* ATCC 25922, *P. aeruginosa* ATCC 10145	Unstudied	Nazir et al., 2024
**Antibody**					
SMAP 28, immunoglobulin G	Unmodified	Covalent coupling	*Porphyromonas gingivalis* strain 381	The antibody targets the outer surface of the bacterium.	Franzman et al., 2009
**Fatty acid chains**					
Anoplin, fatty acid chains	Anoplin with D-amino acid substitutions at positions 4 and 7	Covalent coupling	*E. coli* ATCC 25922 *P. aeruginosa* ATCC 27853 *K. Pneumoniae* ATCC 700603 *A. baumannii* ATCC 19606 *E. coli* ML-35 ATCC 43837 *S. aureus* ATCC 25923 *B. subtilis* ATCC 23857 *S. epidermidis* ATCC 12228	The new peptides exert antimicrobial effects by typical non-receptor-mediated membrane mechanisms as well as intracellular targets.	Zhong et al., 2020
**Photosensitizer**					
PGLa, a π-extended porphyrin photosensitizer	Unmodified	Covalent coupling	*S. aureus*, *E. coli*	Unstudied	Gourlot et al., 2022
HHC10, photosensitizer TPI	Unmodified	Covalent coupling	*E. coli*, *P. aeruginosa*, *S. aureus*, MRSA, *A. baumannii*, *K. pneumoniae*, and *S. pneumoniae*	Antimicrobial peptides target the membrane; photosensitizers generate reactive oxygen species, resulting in synergistic antimicrobial activity.	Fan et al., 2024
**Phosphodiamidate morpholino oligomers (PMO)**					
(RXR)4 XB, PMO target to AcrAB-TolC	Unmodified	Covalent coupling	*P. aeruginosa*	Inhibit expression of efflux pumps and restore susceptibility to antibiotics	Sturge et al., 2019
(RXR)4 XB, PMO target to MCR-1	Unmodified	Covalent coupling	*E. coli*	Enhance cellular entry and target to MCR-1	Daly et al., 2017
(RXR)4 XB/R6G, PMO target to acpP/lpxC/rpsJ	Unmodified	Covalent coupling	*P. aeruginosa*	Target *Pseudomonas* in a gene-specific, species-specific way	Moustafa et al., 2021
**Nanoparticle**					
A synthetic peptide, silver nanoparticle	Unmodified	PEG	*E. coli* ATCC 8739	Unstudied	Gakiya-Teruya et al., 2020
Five CPPs, liposomes	Unmodified	Covalent coupling	*P. aeruginosa*, *E. coli*, MRSA	Induce the liposome-cell membrane fusion and subsequent release of AMPs	Shao et al., 2023
PGLa, glutathione conjugated carbon nanotube (CNT)	Unmodified	Covalent coupling	*E. coli* O157:H7	CNT-bridged 3D graphene oxide helps to trap *E. coli* and allow PGLa to bind easily with *E. coli* O157:H7 bacteria.	Nellore et al., 2015
P9, poly (ethylene glycol)	Stapled Ac-P9	Covalent coupling	*K. pneumoniae*	Unstudied	Cui et al., 2024

(RXR)4 XB/R6G: R is arginine, X is 6-aminohexanoic acid, B is b-alanine and G is glycine; PEG: P: proline; E: glutamic acid; G:glycine.

### 5.1 Antibiotic-peptide conjugates (APCs)

The synergistic mechanism of AMPs and antibiotics may include: ➀ Improve the uptake of antibiotics. ➁ Promote AMP binding to the cell membrane. ➂ Interfere with bacterial metabolism synergistically (Xiao et al., 2023). The goal of such research is to obtain a single-molecule but multifunctional drug with a broad spectrum of antibiotic activity that can kill antibiotic-resistant strains using different or synergistic mechanisms (Gambato et al., 2023).

Gambato et al. covalently linked tobramycin to PrAMP fragments [Bac7(1–15) and Bac7(1–35)] through cleavable disulfide bonds to form a new type of hybrid antibiotic. The tobramycin-PrAMP conjugates effectively inhibited *E. coli* and all seven *P. aeruginosa* strains, with MIC values ranging from 1 to 4 μM. *P. aeruginosa* strains PA10 and PA21 were resistant to both non-conjugated peptides and tobramycin (MIC ≥ 32 μM), but became highly sensitive to conjugates (MIC 2 and 4 μM, respectively). While the mechanism of action of the conjugate remains unclear, given that the components are ineffective individually, the results are highly encouraging and suggest that this could be a means of re-sensitizing antibiotic-resistant pathogens (Gambato et al., 2023). Deshayes et al. (2017) combined CPP (penetratin) with tobramycin to develop new membrane active antibiotic peptide conjugates (MAAPCs). The conjugant successfully integrated the membrane activity of AMP and the function of antibiotics, demonstrating permeability in an outer membrane permeability assay and restoring tobramycin sensitivity in *E. coli* and *S. aureus* persister cells. Notably, the conjugant also has an increased molar mass, which may facilitate its cellular penetration due to a potential decrease in its efflux rate. Some studies suggest that efflux pump efficiency decreases with increasing molecular weight, for example, RND and other multidrug efflux pumps primarily efflux small to medium-sized antibiotics with molecular weights of approximately 300–600 Da (Deshayes et al., 2017; Li et al., 2015). Ghaffar et al. (2015) conjugated the broad-spectrum antibiotic levofloxacin to the highly hydrophobic AMP indolicidin via an amide bond or an unstable ester bond and found that the “permanent” modification of levofloxacin (via amide coupling) reduces its antibacterial activity while modification of levofloxacin on its N-termini did not change its activity. However, the IC50 and MIC values of the unconjugated physical mixture of the two are lower than those of levofloxacin or indolicidin alone (Ghaffar et al., 2015). Therefore, the possible conjugation sites in the structure of the antibiotic under study should be determined, taking into account the structures of the peptide and the drug to reduce the risk of decreased antibiotic activity after conjugation with the AMPs.

### 5.2 Antibody-Antimicrobial Conjugates

Antibody-Antimicrobial Conjugates (AACs) are considered to be an effective alternative to antibiotics in the future, in which AACs target pathogenic bacteria precisely based on specific bacterial antibodies. These specific bacterial antibodies mainly target the components on the bacterial cell wall, which can be divided into three categories based on their structural difference: surface proteins, lipopolysaccharides, and teichoic acid (Yu L. et al., 2023). AACs have emerged as a promising approach to improve target specificity and reduce off-target toxicity, which may help delay the development of resistance; however, its long-term efficacy and clinical potential remain to be validated. Franzman et al. (2009) conjugated sheep myeloid antimicrobial peptide (SMAP) 28 to rabbit immunoglobulin G (IgG) antibodies purified from affinity protein G that are specific to the outer surface of *Porphyromonas gingivalis* strain 381. The antibacterial agent can eliminate *P. gingivalis* without harming the normal commensal flora *in vitro* (Franzman et al., 2009).

### 5.3 Other active molecules

In addition to coupling with known antibiotics or antibodies, AMPs can also be coupled with other active molecules, such as fatty acids, photosensitizers.

The connection with fatty acids can promote the ability to form secondary structures when in contact with bacterial membranes, increase hydrophobicity, and block the action area of proteases. Zhong et al. (2020) designed and synthesized a series of new peptides after substitution of D-amino acids at positions 4 and 7 of anoplin and coupling with fatty acid chains of different lengths. When the length of the conjugated fatty acid chain is in the range of 4–12 carbons, the increased hydrophobicity leads to a 4–32 fold decrease in the MIC of the conjugated peptide until an optimal threshold is reached. Conversely, when the fatty acid chain length exceeds 12 carbons, the MIC increases by 4–16 times (Zhong et al., 2020). Dimeric peptides with the same fatty acid chain length exhibited better antibacterial activity than their monomers. When the fatty acid chain length increased to 8/10/12 carbons, the newer dimers showed the best antibacterial activity with MICs ranging from 2 to 16 μM (Zhong et al., 2019).

Coupling with photosensitizers helps AMPs to effectively kill drug-resistant strains because photosensitizers produce reactive oxygen species (ROS) after exposure to specific light. Effective coupled photosensitizers have been shown to be eosin Y, porphyrins, and protoporphyrin (PpIX) (Kang et al., 2022). Conjugated peptide eosin-(KLAKLAK)2 binds to liposomes of bacterial lipid composition and causes liposomal leakage upon irradiation. The eosin moiety of the conjugate mediates bacterial killing and lipid bilayer leakage by generating the ROS singlet oxygen and superoxide while the (KLAKLAK)2 moiety targets the photosensitizer to bacterial lipid bilayers (Johnson et al., 2014).

Phosphodiamidate morpholino oligomers (PMOs) act as antisense molecules that specifically bind complementary target mRNA and inhibit translation. PMOs have a neutral backbone and cannot efficiently enter bacterial cells. Conjugating PMOs to CPPs increases the delivery of PMOs into cells. Recent data suggest that peptide-conjugated phosphorodiamidate morpholino oligomers (PPMOs) remain active in the context of multidrug-resistant (MDR) strains (Schafer et al., 2021). Moustafa et al. (2021) used targeted genes essential for bacterial growth, including acpP, rpsJ, and lpxC, to prevent the formation of the target proteins, inhibited the *in vitro* growth of several multidrug-resistant clinical *P. aeruginosa* isolates, and demonstrated that alone or in combination with clinically relevant antibiotics, it was effective in reducing biofilm and protecting mice in a lethal model of acute pneumonia. MCR-1 is a plasmid-mediated resistance mechanism to polymyxins. MCR-1 is currently found in Gram-negative organisms that already have a high degree of resistance mechanisms. Daly et al. (2017) described PPMOs that can directly fight bacteria, target genes essential for bacterial growth or block MCR-1, thereby restoring polymyxin sensitivity. The AcrAB-TolC efflux pump has been implicated in resistance to a number of important antibiotic classes including fluoroquinolones, macrolides, and β-lactams (Sturge et al., 2019). PPMOs targeting the genetic components of the AcrABTolC pump were able to reduce *E. coli* resistance to levofloxacin and azithromycin. After PPMO treatment, 2 out of 6 azithromycin-resistant strains (≥32 μg/ml) became sensitive (≤16 μg/ml). In 7 levofloxacin-resistant strains (≥8 μg/ml), 4 out of 7 had their MICs reduced to the intermediate/sensitive range (Sturge et al., 2019).

### 5.4 Nanoparticles

Incorporating AMPs into nanoparticles can combine the advantages of these two biomolecules and overcome their disadvantages (Gan et al., 2021). AMPs can be easily linked or encapsulated into nanodelivery carriers by covalent and non-covalent methods.

Nanocarriers are classified as follows: ➀ Metallic Nanoparticles (MNPs): Mainly divided into gold, silver, and copper oxide nanomaterials, when MNPs interact with bacteria, they can damage the integrity of the cell wall and enter the cytoplasm. By releasing ions in the bacterial environment, MNPs disrupt the respiratory chain mechanism and further increase the permeability of bacterial cells. MNPs can also stimulate oxidative stress and the production of ROS, inhibit ATP production and bacterial DNA replication, and ultimately lead to bacterial death (Makowski et al., 2019). ➁ Polymeric Nanocarriers: PLGA (polylactic-co-glycolic acid) nanoparticles are suitable for sustained-release delivery of AMPs to improve antibacterial effects. Chitosan nanoparticles is a naturally abundant polymer that is non-toxic, biocompatible, biodegradable, and has inherent antibacterial activity due to electrostatic interactions that lead to cell membrane disruption (Imperlini et al., 2023). Hyaluronic acid nanoparticles is a component of the extracellular matrix and possesses high biocompatibility (Sowers et al., 2023). ➂ Liposomes: Liposomes can improve the biocompatibility of AMPs and reduce cytotoxicity, fuse bacterial membranes to enhance antibacterial activity, and protect AMPs from protease degradation. They mainly include liposomes, solid lipid nanoparticles (SLNs), nanostructured lipid carriers (NLCs), and liquid crystal nanoparticles (LCNPs) (Makowski et al., 2019). ➃ Carbon-Based Nanomaterials: Dendrimers are hyperbranched polymer molecules that have been shown to be effective against biofilms, multidrug-resistant bacteria, and viruses (Sowers et al., 2023). Carbon Nanotubes (CNTs) and Graphene Quantum Dots (GQDs) are suitable for AMPs loading to improve cellular uptake and bioavailability (Sowers et al., 2023).

The application of nanotechnology in the field of AMPs can bring many advantages, such as confining AMPs in a rigid conformation of supramolecular scaffolds, increasing the density of positively charged and hydrophobic amino acids, improving antimicrobial activity, and avoiding interactions with salts and proteases. Controlling the accumulation and release of AMPs in specific areas can avoid toxicity and improve targeting (Tan et al., 2021). However, carbon nanotubes are expensive to synthesize and have poor solubility. The drug loading efficiency and immunogenicity of liposomes are a challenge. Disadvantages of polymer nanoparticles include low cell affinity and toxic byproducts (Fadaka et al., 2021). As for gold and silver, the effects of their deposition in the human body require long-term toxicity studies (Makowski et al., 2019). Therefore, further research is needed on the physiological disorders and adverse immune responses of animals after exposure to AMP-NP complexes to overcome the current obstacles that hinder their clinical application (Gonçalves et al., 2022). The preparation process may change the morphology of the peptides and their activity, and the encapsulated peptides may also interact with the walls of the nanocarriers in the nanoenvironment, resulting in incomplete release (Biswaro et al., 2018). How to extend the shelf life of nanomedicines is also a problem. The cost of nanomaterials and large-scale production are one of the obstacles to the effective implementation of these products (Imperlini et al., 2023; Teixeira et al., 2020).

### 5.5 More ideas for conjugation

Adjuvants of antibiotics include drug-resistance enzyme inhibitors, efflux pump inhibitors, outer membrane permeabilizers, biofilm inhibitors. Studies have shown that conjugates containing antibiotics and adjuvants may solve the problem of inconsistency in pharmacodynamics/pharmacokinetics between the two (Gil-Gil et al., 2025). Whether this can also be extended to the field of AMPs requires further study.

#### 5.5.1 Drug-resistance enzyme inhibitors

In the face of the increasing threat of drug resistance, the combined use of beta-lactamase inhibitors (BLIs) with beta-lactam antibiotics is crucial for treating infections caused by drug-resistant bacteria (Huang and Zhou, 2025), such as aztreonam-avibactam (ATM-AVI) (Al Musawa et al., 2024) and cefepime–taniborbactam (Zhanel et al., 2024). In the future, we can consider developing AMPs coupled with new β-lactamase inhibitors (tanibactam, vilbobactam, avibactam) to improve drug-resistant bacterial infections.

#### 5.5.2 Efflux pump inhibitors

Efflux pumps play a central role in enabling bacteria to develop resistance to multiple antibiotics, for example, the *Neisseria gonorrhoeae* MtrCDE efflux pump extrudes antibiotics and host-produced antimicrobial peptides (Gaurav et al., 2023). The use of efflux pump inhibitors can restore the activity of antibiotics that are ineffective against antibiotic-resistant strains (Gil-Gil et al., 2025). To date, several potent efflux pump inhibitors have been discovered. These efflux pump inhibitors belong to structurally diverse chemical classes like peptidomimetics, piperazines, pyridopyrimidines, and pyranopyrimidines (Gaurav et al., 2023). The conjugation of AMPs and efflux pump inhibitors may enhance bactericidal activity against drug-resistant bacteria.

#### 5.5.3 Outer membrane permeabilizers

Permeabilizers help in disintegration of LPS layer leading to disturbance in lipid fraction of outer membrane (OM) causing increase in efficacy of antibiotics toward its target. For instance, Liproxstatin-1 and MAC-0568743 are the compounds which can interact with LPS and help in the disruption of outer membrane (Aggarwal et al., 2024). Conjugating OM permeabilizers to AMPs might provide additional antimicrobial effects.

#### 5.5.4 Biofilm inhibitors

Antimicrobial peptides themselves can target the bacterial membrane and break down the extracellular matrix, destabilizing the biofilm structure. Moreover, AMPs may interfere with the initial stages of biofilm formation by preventing the adhesion of bacteria to surfaces and promoting cell dispersion in the early stages of biofilm formation (Azeem et al., 2025). Quorum-Sensing Inhibitors (QSIs) can disrupt the coordinated activities of bacterial groups and hinder the formation of biofilms by destroying quorum sensing. QSIs such as baicalin, cinnamaldehyde, hamamelitannin, N-(2-pyrimidyl) butanamide (C11), and furanone C-30 could be used in combination with antibiotics (Azeem et al., 2025). Linking AMPs and QSIs may enhance biofilm disruption.

There are no prior literature reports on the conjugation of the above adjuvants with antimicrobial peptides, but it is worth exploring. The premise of constructing the above-mentioned conjugates should be not to destroy their respective structures, otherwise it will affect the function of each part.

### 5.6 New connectors

New connection strategies should be tested, such as self-immolating linkers that release AMPs or antibiotics at the active site after certain stimulation (Dean et al., 2024; Reinhardt and Neundorf, 2016). Cleavable linkers can be categorized as chemically-cleavable or enzyme-cleavable. For example, enzyme-cleavable linkers can be designed for selective cleavage in lysosomes or designed to be more specifically recognized by enzymes. Disulfide bonds in reduction-sensitive linkers are reduced and broken in highly reducing intracellular environments (such as high concentrations of glutathione), releasing the drug. The success of cleavable linkers depends on their ability to effectively distinguish between cyclic conditions and target cellular conditions (Alas et al., 2021; Bargh et al., 2019). The advantage of uncleavable linkers is that they are unlikely to cleave in the plasma like other linker chemicals, prematurely releasing some of the drug into the plasma before reaching the target site. Chemicals such as succinimidyl sulfides, oximes, and triazoles have been used in uncleavable linkers (Fu et al., 2023).

The choice of which type of connector to use requires a trade-off between stability and release efficiency. Finally, the linker chemical is selected to allow the conjugate sufficient cycling time to reach its target cells. Overall, the peptide-drug conjugates (PDCs) should be stable enough that the peptide, linker, and drug are not cleaved or metabolized before reaching the target cells, and that a sufficient concentration of PDCs reaches the target cells (Alas et al., 2021).

## 6 Limitations and challenges

The success of hybrid or Conjugated peptides is somewhat accidental. Even if a hybrid peptide exhibits all the characteristics of an AMP, such as amphiphilicity, hydrophobicity, and positive charge, it may still lack antimicrobial properties. Conjugated peptides are even more complex; different coupling sites and varying drug loading ratios in nanomaterials lead to highly variable pharmacokinetics, efficacy, and toxicity (see Table 4). Therefore, the design of PDCs faces the problems of long cycles and slow progress.

**TABLE 4:** Limitations of natural antimicrobial peptides, and the potential and bottlenecks of hybrid and coupled peptides.

Limitations of natural AMPs	Potential improvements by hybrid or conjugated AMPs	Clinical bottlenecks of hybrid or conjugated AMPs
Emergence of resistance	Synergistic effects or multi-mechanistic compensation	Risk of cross-resistance under long-term exposure; limited evolutionary studies
Cytotoxicity and hemolysis	Targeted delivery and localized release	Target specificity may be reduced *in vivo*
Proteolytic instability	Nanocarriers or modification of protease-sensitive sites	Complex protease environments and poor translational predictability
Short half-life	Nanocarriers or structural optimization	Different hybrids or conjugates have different half-lives.
High production cost	No significant improvement	High manufacturing and GMP scale-up costs
Rapid renal clearance	Increased molecular weight or carrier conjugation	Unstudied

First, the activity and toxicity of peptides change after hybridization, indicating that structural alterations directly affect biological properties. Conjugation of AMP to other functional moieties holds promise for overcoming the limitations of natural molecules, but the design must be carried out in a rational manner to avoid introducing side effects. For example, lipolysis of AMP increases the molecule’s hydrophobicity, which typically increases its activity, but also increases hemolysis and cytotoxicity. Researchers need to understand the advantages and disadvantages of the strategy, which requires considerable work (Reinhardt and Neundorf, 2016).

Second, peptide synthesis remains complex and costly (Rossino et al., 2023). Long sequences (>50 amino acids), incorporation of unnatural residues, cyclization, or polyethylene glycol (PEG) modification often reduce the yield in solid-phase synthesis (Wang L. et al., 2022). Scaling up to Good Manufacturing Practice (GMP) levels requires re-optimization of coupling efficiency, by-product removal, and solvent recovery, which may also increase environmental and production burdens (Al Musaimi, 2024).

Third, the U.S. Food and Drug Administration (FDA) and European Medicines Agency (EMA) have not yet issued AMP-specific regulatory guidelines. The FDA has published general guidance applicable to synthetic peptides (e.g., Guidance for Industry – ANDAs for Certain Highly Purified Synthetic Peptide Drug Products), but no FDA guidance specifically dedicated to AMPs has been identified. Similarly, the EMA has published a draft Guideline on the Development and Manufacture of Synthetic Peptides, which addresses peptide manufacturing and characterization more broadly but is not an AMP-specific finalized guidance. Recent reviews have highlighted that standardized Antimicrobial Susceptibility Testing (AST) methods and interpretive breakpoints for AMPs have not been established (Mercer et al., 2020). Drug susceptibility testing of AMPs needs to take into account differences in peptides, pathogens, and testing environments. Therefore, researchers need to describe the testing methods in detail and test conventional antibiotics simultaneously as reference values to more accurately predict their therapeutic potential (Meurer et al., 2021).

## 7 Computer-aided design strategy

The number of natural AMPs may be in the hundreds of trillions, so new strategies are needed to promote the discovery and design of new AMPs. Machine learning algorithms are computational methods that train input data, self-configuring to produce desired outputs from training data while maintaining generalizability to unseen data.

Current research has developed ensemble models that combine multiple prediction approaches to evaluate peptide activity. For instance, Yao et al. (2024) proposed a three-stage prediction framework named “AMPActiPred,” employing a cascaded deep forest as the core classification/regression model. This framework not only distinguishes antibacterial peptide (ABPs) from non-ABPs but also predicts target-specific activity against various bacterial species and quantitatively estimates activity levels (e.g., MIC values) (Yao et al., 2024). Other studies have integrated LightGBM (gradient boosting trees) with CNN (convolutional neural networks), where LightGBM processes sequence, structural, and physicochemical properties while CNN analyzes structural features and residue types (Zhong et al., 2024). In addition, there are pipeline models, such as using generative models to first generate potential AMP sequences, then using predictive models to evaluate their antibacterial properties, and finally using toxicity and hemolytic activity prediction tools for screening (Capecchi et al., 2021).

Some studies have classified natural AMPs into seven categories according to their sources, including amphibians, bacteria, mammals, insects, fish, humans and plants, and used machine learning models to achieve efficient identification of various AMPs (Horng et al., 2020). Other research has utilized machine learning approaches to predict the activity of antimicrobial peptides against specific Gram-negative bacteria, such as *E. coli* and *A. baumannii* (Teimouri et al., 2023). Some studies have constructed a hierarchical (two-level) machine learning prediction framework: first determine whether the peptide is an AMP, and then further determine whether it has bactericidal activity against *S. aureus* (Khabaz et al., 2023). It can be seen that increasingly accurate and concentrated prediction results are the current research trend. Another emerging research direction involves integrating proteomics with AMP discovery. For instance, Song et al. generated approximately 43,000 peptide sequences (8–50 amino acids in length) from the mucus proteome of *Crassostrea gigas*, then applied the iAMPCN machine learning model to predict antimicrobial activity, identifying six potential AMPs (Song et al., 2024). Similarly, Torres et al. (2024) extracted 444,054 potential small protein families from 1,773 human intestinal metagenomic datasets and employed the AmPEP machine learning model to predict peptides with potential antibacterial activity. The progress of machine learning in the field of antimicrobial peptides in recent years is listed in Table 5. The websites in the table were accessed on October 16, 2025. More information is provided in the Supplementary Table 2.

**TABLE 5:** Progress of artificial intelligence in the field of AMPs in recent years.

Tool	Description	Model	ACC	MCC	Web	References	Year
**Prediction model**							
CAMPR4	Established independent prediction algorithms for both natural and synthetic AMPs	SVM, RF, ANN	Natural AMP:0.87, synthetic AMP:0.94	–	http://camp.bicnirrh.res.in/	Gawde et al., 2023	2023
AMP0	Supports species-specific AMP prediction.	Zero and few shot learning	–	–	http://ampzero.pythonanywhere.com	Gull and Minhas, 2022	2022
CalcAMP	Supports prediction of activity against Gram-positive bacteria, Gram-negative bacteria, and fungi.	RF, Extra Trees, LightGBM, XGBoost, CatBoost	0.79	0.60	–	Bournez et al., 2023	2023
MLBP	Predicts peptides with anticancer, antidiabetic, antihypertensive, anti-inflammatory, and antimicrobial properties.	CNN-BiGRU	0.71	–	https://github.com/xialab-ahu/MLBP	Tang et al., 2022	2022
iAMPCN	Identifies AMPs and their 22 associated biological functions.	CNN	0.99	0.99	https://github.com/joy50706/iAMPCN/tree/master	Xu et al., 2023	2023
MSS AMP	Predicts antimicrobial activity of peptides against specific microbial strains.	RF, SVM, KNN, RealAdaBoost, Multilayer Perceptron, Dl4jMlpClassifier	–	–	https://dbaasp.org/tools?page=genomeprediction	Vishnepolsky et al., 2022	2022
AMPpred-EL	Predicts the antibacterial potential of a given peptide sequence against target bacterial strains.	LightGBM and logistic regression	–	–	https://figshare.com/articles/software/A_bacteria-specific_machine_learning_study_of_individual_antimicrobial_peptide_activity/22129547	Lv et al., 2022	2022
ABP-Finder	Predicts bacterial Gram-staining type targeted by AMP sequences.	RF	0.8	0.50	–	Ruiz-Blanco et al., 2022	2022
PmxPred	Assesses antibacterial potential of polymyxin-like peptides against Gram-negative bacteria.	GCN, catBoost	0.8	0.56	https://github.com/yanwu20/PmxPred	Wang et al., 2024	2024
AMP-Detector	Combines pretrained protein language models with machine learning for sequence representation.	RF, ExtraTrees, XGBoost, HistGradientBoosting	0.95	0.96	–	Medina-Ortiz et al., 2024	2024
E-CLEAP	Integrates amino acid composition (AAC) and pseudo-amino acid composition (PseAAC) features.	Multilayer perceptron classifier	0.97	–	https://github.com/Wangsicheng52/E-CLEAP	Wang, 2024	2024
AMP-BERT	Suitable for peptide sequences with lengths between 10 and 200 amino acids.	ProtBERT	0.76	–	https://github.com/GIST-CSBL/AMP-BERT	Lee et al., 2022	2023
XGBoost	Identifies peptides active against protozoan parasites.	Decision Tree, RF, SVM, Logistic Regression, and XGBoost	0.97	–	www.soodlab.com/appred	Periwal et al., 2024	2024
LABAMPsGCN	Identifies bacteriocins produced by lactic acid bacteria.	CNN	0.94	–	–	Sun et al., 2022	2022
PTPAMP	Specializes in AMPs derived from plant sources.	SVM			http://www.nipgr.ac.in/PTPAMP/	Jaiswal et al., 2022	2023
Embedded-AMP	Designed for large-scale proteome data analysis.	SVM	–	–	–	Carballo et al., 2023	2023
StaBle-ABPpred	Detects novel ABPs in uncharacterized protein sequences.	biLSTM, attention mechanism, RF, gradient boosting, logistic regression	0.98	0.95	https://stable-abppred.anvil.app	Singh et al., 2022	2022
AMPBenchmark	Supports development and benchmarking of various AMP prediction models.	RF, SVM	–	–	–	Sidorczuk et al., 2022	2022
Multi-CGAN	Generates AMP sequences with multiple biological properties.	CGAN	0.82–0.90	–	https://github.com/hqyu/Multi-CGAN	Yu L. et al., 2023	2024
**Generative models**							
Hydramp	Produces peptides with specific antimicrobial activities.	cVAE	–	–	https://hydramp.mimuw.edu.pl/	Szymczak et al., 2023	2023
FBGAN	Produces peptides with specific antimicrobial activities.	GAN	0.81	–	https://github.com/aretiz/de_novo_design_GAN.git	Zervou et al., 2024	2024

ACC, accuracy; MCC, Matthews Correlation Coefficient; SVM, Support Vector Machine; RF, Random Forest; ANN, Artificial Neural Network; CNN, Convolutional Neural Network; LightGBM, Light Gradient Boosting Machine; XGBoost, Extreme Gradient Boosting; GCN, Graph Convolutional Network; CatBoost, Categorical Boosting; CNN-BiGRU, convolutional neural network layer and bidirectional gated recurrent unit layer; KNN, K-nearest neighbors; ProtBERT, pre-trained bidirectional encoder representation from transformers; biLSTM, bidirectional long-short term memory; CGAN, Conditional Generative Adversarial Network; cVAE, Conditional Variational Autoencoder; GAN, Generative Adversarial Network. “–” means no data.

Artificial intelligence also plays a role in antimicrobial peptide design. Compared with traditional AMP design methods, artificial intelligence methods utilize large amounts of sequence space information from dedicated AMP databases to calculate physicochemical parameters or use pattern recognition methods to identify sets of potentially active amino acid substitutions, thereby performing sequence optimization driven by machine learning. Mishra et al. (2025) improved the antimicrobial properties of citropin 1.1 using sequence space information from over 14,743 functional AMPs, producing a short and potent antistaphylococcal peptide, CIT-8 (13 residues), which can eradicate MRSA and VRSA. Wang et al. (2025) developed an interpretable deep learning model, EvoGradient, that can predict the potency of antimicrobial peptides and modify peptide sequences to produce more effective antimicrobial peptides, similar to computer evolution.

Key challenges in AI-assisted antimicrobial peptide design include: ➀ Limited availability of negative sample data for machine learning training; ➁ Insufficient interpretability of sequence-function relationships at critical sites; ➂ Inadequate prediction capabilities for multi-target functions such as low toxicity, stability, solubility, pathogen specificity, immunomodulation, and absorption, distribution, metabolism, excretion (ADME) properties; ➃ Inconsistent prediction results across different computational tools. In the future, it is necessary to standardize laboratory processes and obtain accurate experimental data for training AI. It is also necessary to integrate with 3D structures, diffusion models, etc., to achieve breakthroughs in design accuracy and efficiency (Yan et al., 2022; Wang G. et al., 2022; Brizuela et al., 2025). The structure of peptides is closely related to their antibacterial activity, and adding structural features can enhance prediction performance. Rosetta and AlphaFold can accurately predict protein structures, and GNN and its variants (such as GAT) are effective tools for encoding structural information. In addition, transfer learning can solve the problem of insufficient data (Zhou et al., 2025).

Currently, the success of hybrid or conjugated peptides depends on experimenter experimentation; there are no universal guidelines, and success is often serendipitous. Perhaps artificial intelligence can advance the development of hybrid or conjugated peptides. For example, omics tools can be used to discover a large number of potential antimicrobial peptides, perform virtual hybridization and ultimately validate them through experimental testing. However, true virtual hybridization algorithms have yet to be established, and existing AI platforms (such as DeepAMP, AMPfun, and HemoPI-2) can only predict the activity/toxicity of single peptides. In addition, Data must be structured, multi-center, and standardized. Rather than simply stacking numbers, unified measurements of MIC, hemolysis, solubility, and pharmacokinetics must be required. Otherwise, machine learning models will not be transferable across laboratories. AI must also consider whether it can be engineered and scaled up for production. The generation of large amounts of standardized experimental data can facilitate machine learning, e.g., determining whether hybridization yields better results and selecting linkers and attachment sites, further facilitating the discovery of a large number of hybrid/conjugated peptides.

## 8 Conclusions and future perspectives

Antimicrobial peptides come from a wide range of sources, and their structural and functional diversity lays a solid foundation for subsequent rationalization. Whether using traditional chemical modification strategies, nanomaterials for conformational synergy, or machine learning for sequence design, the key physicochemical parameters-peptide length, net charge, conformational preference, hydrophobicity, amphiphilicity, and specific amino acid residues-are systematically considered and precisely regulated.

There are approximately 96 Peptide-Drug Conjugates (PDCs) in the clinical trial phase, most of which are for cancer indications, and Lutathera is currently the only PDC on the market that has been approved by the FDA (He et al., 2025; Armstrong et al., 2025). No hybrid or coupled peptides with antibacterial indications have been under the clinical trial stage. In the future, whether it is possible to adopt the strategy of peptide connecting more than two antibiotics, or the strategy of dual-target peptide + antimicrobial peptide, these are worth further exploration.

## Footnotes

1
https://aps.unmc.edu/AP
