# Supplementary material for: Hybrid and conjugated antimicrobial peptides: new tactics to counter bacterial resistance
Source: Front Microbiol. 2026 Mar 10;17:1670569. doi: 10.3389/fmicb.2026.1670569 (PMC13014047; doi:10.3389/fmicb.2026.1670569)
Supplement: Supplementary file 1 [file Table_1.docx]

**Supplementary Materials**

**Table S1. Examples of different types of hybrid peptides**

| hybridizer | source | modification | linker | antibacterial spectrum | effect | ref. |
| --- | --- | --- | --- | --- | --- | --- |
| Hybridization between two natural antimicrobial peptides | | | | | | |
| Indolicidin,  Ranalexin | cattle,  frog | truncate both parent peptide | directly linked | *Streptococcus pneumoniae* | breach cell walls and inhibited DNA synthesis, reduce the therapeutic concentrations of ceftriaxone | [1] |
| melittin,  thanatin | *Apis mellifera*,  *Podisus maculiventris* | truncate melittin(Ala→Lys substitution) | AGP | *E.coli* JM109,  *S.aureus*,  *Bacillus subtilis*,  *Salmonella Typhimurium* | produce an anti-*S.aureus* effect and reduce hemolysis | [2] |
| VmCT1,  anoplin/protonectin/decoralin/temporin A | *Vaejovis mexicanus*,  *Anoplius samariensis/Agelaia pallipes/Oreumenes decorates/Rana temporaria* | truncate the amphipathic segment of the parent peptide | G | *A.baumannii* ATCC 19606,  *E.coli*( ATCC 11775,AIC221,AIC222 stains),  *Klebsiella pneumoniae* ATCC 13883,  *S.aureus* ATCC 12600,and etc. | redirect biological  activity, present increased antimicrobial activity with almost no adverse toxicity to erythrocytes | [3] |
| papiliocin,  magainin II | *Papilio xuthus*,  *Xenopus laevis* | truncate both parent peptide | P/a lysine peptoid analogue | *B.subtilis* KCTC 3068,  *E. faecalis* KCTC 2011,  *S.aureus* KCTC 1621,  *S.typhi* KCTC 1926,  *P.aeruginosa* KCTC 1637,  *E.coli* KCTC 1682 | have antibacterial and anti-inflammatory activities, PapMA-k display enhanced bacterial selectivity | [4] |
| BMAP-27,  LL-37 | bovine neutrophils,  human | extract the helical portion of the parent peptide | directly linked | *S. aureus* (ATCC 29213,ATCC 33591,ATCC 43300 ,ATCC BAA-41strains),  *S. epidermidis* ATCC12228,  *E. faecium* BAA-2316,  *E. coli* (ATCC 25922,ATCC 35218 strains),  MDR *P.aeruginosa* ATCC BAA-2114 | increased activity, reduced toxicity, and synergistic effect with conventional antibiotics | [5] |
| magainin II,  cecropin B | *Xenopus laevis,*  *Cecropia* *moth*, | - | directly linked | *E.coli* ATCC 25922 | display antibacterial and immunomodulatory activities in mice | [6] |
| LfcinB6,  KR-12-a4 | bovine lactoferrin,  human | - | P | *B.subtilis* KCTC 3068,  *S.epidermalis* KCTC 1917,  *S.aureus* KCTC 1621,  *E.coli* KCTC 1682,  *P.aeruginosa* KCTC 1637,  *S.typhi* KCTC 1926 | exert high cell  selectivity for bacterial cells, have broad-spectrum antimicrobial activity without increasing hemolytic activity | [7] |
| CopA3,  Hp1090 | *Copris tripartitus*,  *Heterometrus petersii* | - | GG/GGGGGG | *E. coli* (D31 and JM83 strains) | have more potent bactericidal activity, adjust the nature of the linker peptide to modulate the cytotoxic activity | [8] |
| LL-37,  Renalexin | human,  frog | - | GS | *S.aureus*, *E.coli*, MRSA, and *K.pneumoniae* bacteria clinical isolates | higher antibacterial potency | [9] |
| nisin(1-20),  Thanatin | *Lactococcus lactis*,  hemipteran insect *Podisus maculiventris* | convert disulfide-bond-based AMPs into hybrid (methyl)lanthionine-based macrocyclic lanthipeptides | directly linked | *S.aureus*,MASA | produce an anti-*S.aureus* effect, have highly resistant against the nisin resistance protein | [10] |
| Melittin, Lasioglossin | *Apis mellifera*, Lasioglossum laticeps venom | truncate melittin(Arg→IIe substitution) | directly linked | *A. baumanni* and *S. aureus* | expand antibacterial spectrum, have high stability | [11] |
| BMAP-27,  Cecropin-A | bovine neutrophils,  insects | truncate both parent peptide,  Cecropin-A: Lys→Asp substitution | directly linked | *S.aureus* ATCC 29213,  MRSA ATCC Baa-41,  *E.coli (*ATCC 25922, ATCC Baa-2452) | show better activity, selectivity, and safety profile | [12] |
| Cathelicidin,  Aurein | vertebrates,  Australian southern bell frogs *Litoria aurea* and *Litoria raniformis* | truncate both parent peptide,  Cathelicidin: Lys→Ala,Gly→Trp,Asp→Leu,Leu→Arg substitution,  Aurein: Gly→Arg,Asp→Arg,IIe→Arg substitution,  truncate hybrid peptide | directly linked | MDR *P.aeruginosa* | have higher antimicrobial activity with reduced hemolytic activity | [13] |
| BMAP-28,  LL-37 | bovine neutrophils,  human | extract the helical portion of the parent peptide,  BMAP-28:Arg3→Lys3, Lys14→Arg14 substitution,  LL-37:Phe→Ala,Lys→Val substitution | directly linked | MRSA,  MDR *E. coli*,  *S. aureus* (29213 and BAA-41 stains) | have higher antimicrobial activity with reduced toxicity | [14] |
| BF2,  DesHDAP1 | histone-derived AMPs | - | A/P/G/hydroxyalanine | *E. coli*, *B. subtilis* | have higher antimicrobial activity with no cytotoxicity against eukaryotic cells | [15] |
| enterocinK ,  EJ97 | Bacteriocins | truncate both parent peptide | YEI | *Staphylococcus haemolyticus* | differ from other bacteriocins in the inhibition spectrum | [16] |
| cesin,  rombocin | Nisin | truncate both parent peptide | directly linked | *Lactococcus lactis* MG1363,  *Listeria monocytogenes* LK132,  *Bacillus cereus* CH-85,  *S. aureus* LMG10147,  MASA,  *E.faecium(*LMG11423,LMG16003 strains) | maintain high efficiency, significantly improve resistance to pancreatic enzyme degradation, show no undesired toxic effects, remain stable in human plasma | [17] |
| dermorphin, ranatensin | *Phyllomedusa sauvagei*,  *Rana pipiens* | - | directly linked | *E. coli* strains K12 and R1-R4 | have superior activity compared with ciprofloxacin or bleomycin | [18] |
| papiliocin,  magainin II | *Papilio xuthus*,  *Xenopus laevis* | truncate both parent peptide,  magainin II: Ala15,Phe18→Ala,Phe,and Trp | P | Carbapenem-resistant Acinetobacter baumannii (CRAB) | demonstrate potent  anti-microbial activity against CRAB without notable cytotoxicity, have synergistic effect with rifampin | [19] |
| LL-37,  human beta-defensin-1 to -3 | human | truncate both parent peptide | directly linked | *S. aureus* ATCC SA29213 | potentially effective and safe topical antimicrobial agents with no/minimal risk of bacterial resistance. | [20] |
| Thymosin | | | | | | |
| LL-37(13-36)/YW12D/innate defense regulator 1/cathelicidin 2,  TP5/Tα1(17-24) | human/synthesis/  chicken  synthesis/ thymic stromal cells | truncate both parent peptide | directly linked | bind to LPS | enhanced anti-inflammatory activity and reduced cytotoxicity | [21] |
| cathelicidin 2,  TP5 | chicken  synthesis | truncate both parent peptide, add Gln to the C-terminus | directly linked | *S.aureus* ATCC 43300 | have effective antibacterial, antibiofilm, and anti-adhesion activities | [22] |
| New amyloidogenic antimicrobial peptides | | | | | | |
| CPP,  Amyloidogenic Sequence of Ribosomal S1 Protein of P. aeruginosa |  | sarcosine12 instead of Gly12 | GGGG,GG-Sar-G | *P. aeruginosa*（PA103 and ATCC 28753 stains） | The MIC was comparable to the antibiotic gentamicin sulfate. | [23] |
| CPP,  Amyloidogenic Sequence of Ribosomal S1 Protein from S. aureus |  | Asi19 instead of Asn19, sarcosine instead of Gly | GG-Sar-G | MRSA (ATCC 43300 strain),  *S.aureus* 209P,  *E.coli* K12 strain,  *B.cereus* IP-5812 strain | active against Gram-positive and Gram-negative bacteria | [24] |
| CPP,  Amyloidogenic Sequence of Ribosomal S1 Protein from S. aureus |  | replacement of canonical amino acid residues with other canonical or non-canonical amino acid residues (X) | GG-Sar-G | *S.aureus* (209P and 129B strains),  MRSA(SA 180 and ATCC 43300 strains),  *B.cereus* (strain IP 5832),  *P.aeruginosa* (ATCC 28753 and 2943 strains)  *E.coli* (MG1655 and K12 strains) | exhibit antimicrobial activity comparable to gentamicin and meropenem | [25] |
| Targeting peptides | | | | | | |
| an LPS binding sequence,  PMAP-23 |  | Arg instead of Asp | PQKP | *E.coli* ATCC 25922,  *S.aureus*(ATCC 25923 and 29213 strains),  *S.typhi* C7731,  *S.epidermidis* ATCC 12228 | enhance LPS affinity | [26] |
| A targeting peptide binds to *P.aeruginosa*,  GNU7 |  | - | GGG | MDR *P.aeruginosa* | exhibit a high degree of specificity for P. aeruginosa | [27] |

A: alanine; G: glycine; P: proline; S: serine; Y: Tyrosine; E: glutamic acid; I: isoleucine; Q: glutamine; K: lysine

**Table S2: Progress of artificial intelligence in the field of AMPs in recent years**

| Tool | Describe | Model | ACC | MCC | Web | Ref. | Year |
| --- | --- | --- | --- | --- | --- | --- | --- |
| Prediction Model | | | | | | | |
| APD3 | Currently focus on natural AMPs with defined sequences and validated activities. | Peptide Parameter Space |  |  | <https://aps.unmc.edu/AP> | [28] | 2016 |
| CAMPR4 | Established independent prediction algorithms for both natural and synthetic AMPs | SVM,RF,ANN | natural AMP:0.87,synthetic AMP:0.94 |  | <http://camp.bicnirrh.res.in/> | [29] | 2023 |
| dbAMP | Supports species-specific AMP prediction. | RF,BLASTP | 0.93 | - | <http://csb.cse.yzu.edu.tw/dbAMP/> | [30] | 2020 |
| AMP0 | Supports species-specific AMP prediction. | Zero and Few Shot Learning | - | - | <http://ampzero.pythonanywhere.com> | [31] | 2022 |
| AMPScanner | Applies deep learning techniques for AMP sequence classification. | CNN,LSTM | 0.90 | 0.83 | <https://www.dveltri.com/ascan/v2/ascan.html> | [32] | 2018 |
| AMPfun | Characterizes sequence features of AMPs with activity against parasites, viruses, cancer cells, fungi, and both Gram-positive and Gram-negative bacteria in mammals. | DT,RF,SVM | 0.88 | 0.76 | <http://fdblab.csie.ncu.edu.tw/AMPfun/index.html> | [33] | 2019 |
| iAMPpred | Serves as a supplement to existing predictors for antibacterial, antiviral, and antifungal peptides. | SVM | 0.89 | 0.89 | [http://www.cabgrid.res.cn/8080/amppred /](http://www.cabgrid.res.cn/8080/amppred%20/) | [34] | 2019 |
| CalcAMP | Supports prediction of activity against Gram-positive bacteria, Gram-negative bacteria, and fungi. | RF,Extra Trees（ET）,LightGBM,XGBoost,CatBoost | 0.79 | 0.60 | <https://doi.org/10.5281/zenodo.7588702> | [35] | 2023 |
| iAMP-CA2L | Covers AMPs with antibacterial, antiviral, antifungal, anti-biofilm, anti-parasitic, anti-HIV, anticancer, chemotactic, anti-MRSA, and anti-endotoxin activities. | CNN,BiLSTM,SVM | 0.96 | 0.92 | <http://www.jci-bioinfo.cn/iAMP-CA2L> | [36] | 2021 |
| MLBP | Predicts peptides with anticancer, antidiabetic, antihypertensive, anti-inflammatory, and antimicrobial properties. | CNN-BiGRU | 0.71 | - | <https://github.com/xialab-ahu/MLBP> | [37] | 2022 |
| iAMPCN | Identifies AMPs and their 22 associated biological functions. | CNN | 0.99 | 0.99 | <https://github.com/joy50706/iAMPCN/tree/master> | [38] | 2023 |
| MSS AMP | Predicts antimicrobial activity of peptides against specific microbial strains. | RF,LibSVM,KNN,RealAdaBoost,Multilayer Perceptron (Neuron network),Dl4jMlpClassifier | - | - | <https://dbaasp.org/tools?page=genomeprediction> | [39] | 2022 |
| AMPpred-EL | Predicts the antibacterial potential of a given peptide sequence against target bacterial strains. | LightGBM and logistic regression | - | - | <https://figshare.com/articles/software/A_bacteria-specific_machine_learning_study_of_individual_antimicrobial_peptide_activity/22129547> | [40] | 2022 |
| ABP-Finder | Predicts bacterial Gram-staining type targeted by AMP sequences. | RF | 0.8 | 0.50 | <https://protdcal.zmb.uni-due.de/ABP-Finder/> | [41] | 2022 |
| PmxPred | Assesses antibacterial potential of polymyxin-like peptides against Gram-negative bacteria. | GCN,catBoost | 0.8 | 0.56 | <https://github.com/yanwu20/PmxPred> | [42] | 2024 |
| AMP-Detector | Combines pretrained protein language models with machine learning for sequence representation. | RF,ExtraTrees,XGBoost,HistGradientBoosting | 0.95 | 0.96 | <https://drive.google.com/drive/folders/1IO_mL6Jf7vGQZ6aE7IK6crQFiLzZ62Cf> | [43] | 2024 |
| IAMPE | Predicts AMP activity based solely on sequence data. | NB,KNN,SVM,RF,XGBoost | 0.95 | - | <http://cbb1.ut.ac.ir/> | [44] | 2020 |
| E-CLEAP | Integrates amino acid composition (AAC) and pseudo-amino acid composition (PseAAC) features. | Multilayer Perceptron Classifier | 0.97 | - | <https://github.com/Wangsicheng52/E-CLEAP> | [45] | 2024 |
| Deep-AmPEP30 | Limited to peptides shorter than 30 amino acids in length. | CNN,RF | 0.77 | 0.54 | <https://cbbio.cis.um.edu.mo/AxPEP> | [46] | 2020 |
| DBAASP | Only applicable to peptides shorter than 100 amino acids. | New algorithm DBSCAN | - | - | <https://dbaasp.org> | [47] | 2020 |
| AniAMPpred | Recognizes peptide sequences ranging from 10 to 200 amino acids in animal genomes. | SVM,DNN | 0.97 | - | <https://aniamppred.anvil.app/> | [48] | 2021 |
| AMP-BERT | Suitable for peptide sequences with lengths between 10 and 200 amino acids. | ProtBERT-BFD | 0.76 | - | <https://github.com/GIST-CSBL/AMP-BERT> | [49] | 2023 |
| XGBoost | Identifies peptides active against protozoan parasites. | Decision Tree,RF, SVM, Logistic Regression, and XGBoost | 0.97 | - | [www.soodlab.com/appred](http://www.soodlab.com/appred) | [50] | 2024 |
| LABAMPsGCN | Identifies bacteriocins produced by lactic acid bacteria. | GCN | 0.94 | - | <http://www.dong-group.cn/database/dlabamp/Prediction/amplab/result/> | [51] | 2022 |
| PTPAMP | Specializes in AMPs derived from plant sources. | SVM |  |  | <http://www.nipgr.ac.in/PTPAMP/> | [52] | 2023 |
| DefPred | Predicts defensins. | SVM | 0.98 | 0.96 | <https://webs.iiitd.edu.in/raghava/defpred> | [53] | 2021 |
| Embedded-AMP | Designed for large-scale proteome data analysis. | SVM | - | - | <https://biocom-ampdiscover.cicese.mx> | [54] | 2023 |
| AmpGram | Predicts and designs AMPs based on proteomics data. | RF | - | - | <http://biongram.biotech.uni.wroc.pl/AmpGram/> | [55] | 2020 |
| Deep-ABPpred | Identifies antibacterial peptides (ABPs) within protein sequences. | BiLSTM,word2vec | 0.96 | 0.91 | <https://abppred.anvil.app/> | [56] | 2021 |
| StaBle-ABPpred | Detects novel ABPs in uncharacterized protein sequences. | biLSTM,attention mechanism,RF,gradient boosting,logistic regression | 0.98 | 0.95 | <https://stable-abppred.anvil.app> | [57] | 2022 |
| amPEPpy | Enables AMP sequence mining from genome-scale datasets | RF | 0.87 | 0.88 | <https://github.com/tlawrence3/amPEPpy> | [58] | 2021 |
| AMPBenchmark | Supports development and benchmarking of various AMP prediction models. | RF,SVM, | - | - | <http://BioGenies.info/AMPBenchmark> | [59] | 2022 |
| Generative Models | | | | | | | |
| Multi-CGAN | Generates AMP sequences with multiple biological properties. | CGAN | 0.82-0.90 | - | <https://github.com/hqyu/Multi-CGAN> | [60] | 2024 |
| hydramp | Produces peptides with specific antimicrobial activities. | cVAE | - | - | <https://hydramp.mimuw.edu.pl/> | [61] | 2023 |
| FBGAN | Produces peptides with specific antimicrobial activities. | GAN | 0.81 | - | <https://github.com/aretiz/de_novo_design_GAN.git> | [62] | 2024 |

ACC, Accuracy; MCC, Matthews Correlation Coefficient; SVM, Support Vector Machine; RF, Random Forest; ANN, Artificial Neural Network; CNN, Convolutional Neural Network; GAN, Generative Adversarial Network; cVAE, Conditional Variational Autoencoder.

.

Reference:

1. Jindal, H.M., et al., *Mechanisms of action andin vivoantibacterial efficacy assessment of five novel hybrid peptides derived from Indolicidin and Ranalexin against Streptococcus pneumoniae.* PeerJ, 2017. **5**.

2. Jiang, X., et al., *Design and activity study of a melittin–thanatin hybrid peptide.* AMB Express, 2019. **9**(1).

3. Pedron, C.N., et al., *Molecular hybridization strategy for tuning bioactive peptide function.* Communications Biology, 2023. **6**(1).

4. Shin, A., et al., *Peptoid-Substituted Hybrid Antimicrobial Peptide Derived from Papiliocin and Magainin 2 with Enhanced Bacterial Selectivity and Anti-inflammatory Activity.* Biochemistry, 2015. **54**(25): p. 3921-3931.

5. Al Tall, Y., et al., *Design and characterization of a new hybrid peptide from LL-37 and BMAP-27.* Infection and Drug Resistance, 2019. **Volume 12**: p. 1035-1045.

6. Zhang, M., et al., *Expression of a recombinant hybrid antimicrobial peptide magainin II-cecropin B in the mycelium of the medicinal fungus Cordyceps militaris and its validation in mice.* Microbial Cell Factories, 2018. **17**(1).

7. Ajish C, Yang S, Kumar SD, et al.*,A novel hybrid peptide composed of LfcinB6 and KR-12-a4 with enhanced antimicrobial, anti-inflammatory and anti-biofilm activities.* Sci Rep. 2022;12(1):4365.

8. Tonk, M., et al., *Potent Activity of Hybrid Arthropod Antimicrobial Peptides Linked by Glycine Spacers.* Int J Mol Sci, 2021. **22**(16).

9. Narh, J.K., N.G. Casillas-Vega, and X. Zarate, *LL-37_Renalexin hybrid peptide exhibits antimicrobial activity at lower MICs than its counterpart single peptides.* Appl Microbiol Biotechnol, 2024. **108**(1): p. 126.

10. Zhao, X. and O.P. Kuipers, *Nisin- and Ripcin-Derived Hybrid Lanthipeptides Display Selective Antimicrobial Activity against Staphylococcus aureus.* ACS Synth Biol, 2021. **10**(7): p. 1703-1714.

11. Nabizadeh, S., et al., *Rational design of hybrid peptide with high antimicrobial property derived from Melittin and Lasioglossin.* J Biomol Struct Dyn, 2024. **42**(23): p. 13091-13099.

12. Masadeh, M.M., et al., *The Antimicrobial Effect Against Multi-drug Resistant Bacteria of the SK4 Peptide: A Novel Hybrid Peptide of Cecropin-A and BMAP-27.* Curr Pharm Biotechnol, 2023. **24**(8): p. 1070-1078.

13. Klubthawee, N., et al., *A novel, rationally designed, hybrid antimicrobial peptide, inspired by cathelicidin and aurein, exhibits membrane-active mechanisms against Pseudomonas aeruginosa.* Sci Rep, 2020. **10**(1): p. 9117.

14. Masadeh, M., et al., *Functional and Toxicological Evaluation of MAA-41: A Novel Rationally Designed Antimicrobial Peptide Using Hybridization and Modification Methods from LL-37 and BMAP-28.* Curr Pharm Des, 2022. **28**(26): p. 2177-2188.

15. Trevellin, G.F., et al., *Hybrids of Membrane-Translocating Antimicrobial Peptides Show Enhanced Activity through Membrane Permeabilization.* ACS Med Chem Lett, 2024. **15**(11): p. 1918-1924.

16. Kranjec, C., et al., *A bacteriocin-based treatment option for Staphylococcus haemolyticus biofilms.* Scientific Reports, 2021. **11**(1).

17. Guo, L., et al., *Engineering hybrid lantibiotics yields the highly stable and bacteriocidal peptide cerocin V.* Microbiol Res, 2024. **282**: p. 127640.

18. Serafin, P., et al., *Evaluation of Antimicrobial Activities against Various E. coli Strains of a Novel Hybrid Peptide-LENART01.* Molecules, 2023. **28**(13).

19. Choi, J., et al., *Development of Novel Peptides for the Antimicrobial Combination Therapy against Carbapenem-Resistant Acinetobacter baumannii Infection.* Pharmaceutics, 2021. **13**(11).

20. Ting, D.S.J., et al., *Hybrid derivative of cathelicidin and human beta defensin-2 against Gram-positive bacteria: A novel approach for the treatment of bacterial keratitis.* Sci Rep, 2021. **11**(1): p. 18304.

21. Zhang, L., et al., *Design and Development of a Novel Peptide for Treating Intestinal Inflammation.* Frontiers in Immunology, 2019. **10**.

22. Guo, H.N., et al., *Novel Hybrid Peptide Cathelicidin 2 (1-13)-Thymopentin (TP5) and Its Derived Peptides with Effective Antibacterial, Antibiofilm, and Anti-Adhesion Activities.* Int J Mol Sci, 2021. **22**(21).

23. Grishin, S.Y., et al., *Is It Possible to Create Antimicrobial Peptides Based on the Amyloidogenic Sequence of Ribosomal S1 Protein of P. aeruginosa?* International Journal of Molecular Sciences, 2021. **22**(18).

24. Kravchenko, S.V., et al., *Multiple Antimicrobial Effects of Hybrid Peptides Synthesized Based on the Sequence of Ribosomal S1 Protein from Staphylococcus aureus.* International Journal of Molecular Sciences, 2022. **23**(1).

25. Kravchenko, S.V., et al., *Optimizing Antimicrobial Peptide Design: Integration of Cell-Penetrating Peptides, Amyloidogenic Fragments, and Amino Acid Residue Modifications.* Int J Mol Sci, 2024. **25**(11).

26. Lyu, Y., et al., *Broad-spectrum hybrid antimicrobial peptides derived from PMAP-23 with potential LPS binding ability.* Biochemical Pharmacology, 2023. **210**.

27. Kim, H., et al., *Development of a novel hybrid antimicrobial peptide for targeted killing of Pseudomonas aeruginosa.* Eur J Med Chem, 2020. **185**: p. 111814.

28. Wang, G., X. Li, and Z. Wang, *APD3: the antimicrobial peptide database as a tool for research and education.* Nucleic Acids Research, 2016. **44**(D1): p. D1087-D1093.

29. Gawde, U., et al., *CAMPR4: a database of natural and synthetic antimicrobial peptides.* Nucleic Acids Research, 2023. **51**(D1): p. D377-D383.

30. Jhong, J.-H., et al., *dbAMP: an integrated resource for exploring antimicrobial peptides with functional activities and physicochemical properties on transcriptome and proteome data.* Nucleic Acids Research, 2019. **47**(D1): p. D285-D297.

31. Sadaf Gull, F.M., *AMP0: Species-Specific Prediction of Antimicrobial Peptides using Zero and Few Shot Learning.* IEEE/ACM transactions on computational biology and bioinformatics, 2022.

32. Veltri, D., et al., *Deep learning improves antimicrobial peptide recognition.* Bioinformatics, 2018. **34**(16): p. 2740-2747.

33. Horng, J.-T., et al., *Characterization and identification of antimicrobial peptides with different functional activities.* Briefings in Bioinformatics, 2020. **21**(3): p. 1098-1114.

34. Meher, P.K., et al., *Predicting antimicrobial peptides with improved accuracy by incorporating the compositional, physico-chemical and structural features into Chou’s general PseAAC.* Scientific Reports, 2017. **7**(1).

35. Bournez, C., et al., *CalcAMP: A New Machine Learning Model for the Accurate Prediction of Antimicrobial Activity of Peptides.* Antibiotics, 2023. **12**(4).

36. Xiao, X., et al., *iAMP-CA2L: a new CNN-BiLSTM-SVM classifier based on cellular automata image for identifying antimicrobial peptides and their functional types.* Briefings in Bioinformatics, 2021. **22**(6).

37. Tang, W., et al., *Identifying multi-functional bioactive peptide functions using multi-label deep learning.* Briefings in Bioinformatics, 2022. **23**(1).

38. Xu, J., et al., *iAMPCN: a deep-learning approach for identifying antimicrobial peptides and their functional activities.* Briefings in Bioinformatics, 2023. **24**(4).

39. Vishnepolsky, B., et al., *Comparative analysis of machine learning algorithms on the microbial strainspecific AMP prediction.* 2022.

40. Lv, H., et al., *AMPpred-EL: An effective antimicrobial peptide prediction model based on ensemble learning.* Computers in Biology and Medicine, 2022. **146**.

41. Ruiz-Blanco, Y.B., et al., *ABP-Finder: A Tool to Identify Antibacterial Peptides and the Gram-Staining Type of Targeted Bacteria.* Antibiotics, 2022. **11**(12).

42. Wang, X., et al., *PmxPred: A data-driven approach for the identification of active polymyxin analogues against gram-negative bacteria.* Computers in Biology and Medicine, 2024. **168**.

43. Medina-Ortiz, D., et al., *Protein Language Models and Machine Learning Facilitate the Identification of Antimicrobial Peptides.* International Journal of Molecular Sciences, 2024. **25**(16).

44. !!! INVALID CITATION !!! [157].

45. Akbar, S. and S.-C. Wang, *E-CLEAP: An ensemble learning model for efficient and accurate identification of antimicrobial peptides.* Plos One, 2024. **19**(5).

46. Yan, J., et al., *Deep-AmPEP30: Improve Short Antimicrobial Peptides Prediction with Deep Learning.* Molecular Therapy - Nucleic Acids, 2020. **20**: p. 882-894.

47. Pirtskhalava, M., et al., *DBAASP v3: database of antimicrobial/cytotoxic activity and structure of peptides as a resource for development of new therapeutics.* Nucleic Acids Research, 2021. **49**(D1): p. D288-D297.

48. Sharma, R., et al., *AniAMPpred: artificial intelligence guided discovery of novel antimicrobial peptides in animal kingdom.* Briefings in Bioinformatics, 2021. **22**(6).

49. Lee, H., et al., *AMP‐BERT: Prediction of antimicrobial peptide function based on a BERT model.* Protein Science, 2022. **32**(1).

50. Periwal, N., et al., *Antiprotozoal peptide prediction using machine learning with effective feature selection techniques.* Heliyon, 2024. **10**(16).

51. Sun, T.-J., et al., *LABAMPsGCN: A framework for identifying lactic acid bacteria antimicrobial peptides based on graph convolutional neural network.* Frontiers in Genetics, 2022. **13**.

52. Jaiswal, M., A. Singh, and S. Kumar, *PTPAMP: prediction tool for plant-derived antimicrobial peptides.* Amino Acids, 2022. **55**(1): p. 1-17.

53. Kaur, D., et al., *In-Silico Tool for Predicting, Scanning, and Designing Defensins.* Frontiers in Immunology, 2021. **12**.

54. Carballo, G.M., et al., *Embedded-AMP: A Multi-Thread Computational Method for the Systematic Identification of Antimicrobial Peptides Embedded in Proteome Sequences.* Antibiotics, 2023. **12**(1).

55. !!! INVALID CITATION !!! [168].

56. Sharma, R., et al., *Deep-ABPpred: identifying antibacterial peptides in protein sequences using bidirectional LSTM with word2vec.* Briefings in Bioinformatics, 2021. **22**(5).

57. Singh, V., et al., *StaBle-ABPpred: a stacked ensemble predictor based on biLSTM and attention mechanism for accelerated discovery of antibacterial peptides.* Briefings in Bioinformatics, 2022. **23**(1).

58. Lawrence, T.J., et al., *amPEPpy 1.0: a portable and accurate antimicrobial peptide prediction tool.* Bioinformatics, 2021.

59. Sidorczuk, K., et al., *Benchmarks in antimicrobial peptide prediction are biased due to the selection of negative data.* Briefings in Bioinformatics, 2022. **23**(5).

60. Yu, H., et al., *Multi-CGAN: Deep Generative Model-Based Multiproperty Antimicrobial Peptide Design.* Journal of Chemical Information and Modeling, 2023. **64**(1): p. 316-326.

61. Szymczak, P., et al., *Discovering highly potent antimicrobial peptides with deep generative model HydrAMP.* Nature Communications, 2023. **14**(1).

62. Zervou, M., et al., *De Novo Antimicrobial Peptide Design with Feedback Generative Adversarial Networks.* International Journal of Molecular Sciences, 2024. **25**(10).
